# Biomimetic Photodegradation of Glyphosate in Carborane-Functionalized Nanoconfined Spaces

**DOI:** 10.1021/jacs.3c02019

**Published:** 2023-06-20

**Authors:** Lei Gan, Makenzie T. Nord, Jacob M. Lessard, Noah Q. Tufts, Arunraj Chidambaram, Mark E. Light, Hongliang Huang, Eduardo Solano, Julio Fraile, Fabián Suárez-García, Clara Viñas, Francesc Teixidor, Kyriakos C. Stylianou, José G. Planas

**Affiliations:** † Institut de Ciència de Materials de Barcelona (ICMAB-CSIC), Bellaterra 08193, Spain; ‡ Institute of Physical and Theoretical Chemistry, Graz University of Technology, Graz 8010, Austria; § Materials Discovery Laboratory (MaD Lab), Department of Chemistry, Oregon State University, 153 Gilbert Hall, Corvallis, Oregon OR 97331, United States; ∥ Institute of Chemical Sciences and Engineering, École Polytechnique Fedérale de Lausanne (EPFL Valais), Rue de l’Industrie 17, Sion 1951, Switzerland; ⊥ Chemspeed Technologies AG, Wölferstrasse 8, Füllinsdorf 4414, Switzerland; # Department of Chemistry, University of Southampton, Highfield, Southampton SO17 1BJ, U.K.; ¶ State Key Laboratory of Separation Membranes and Membrane Processes, School of Chemistry and Chemical Engineering, Tiangong University, Tianjin 300387, China; ∇ NCD-SWEET Beamline, ALBA Synchrotron Light Source, Cerdanyola del Vallès 08290, Spain; ○ Departament of Material Chemistry, Instituto de Ciencia y Tecnología del Carbono, INCAR-CSIC, Oviedo 33011, Spain

## Abstract

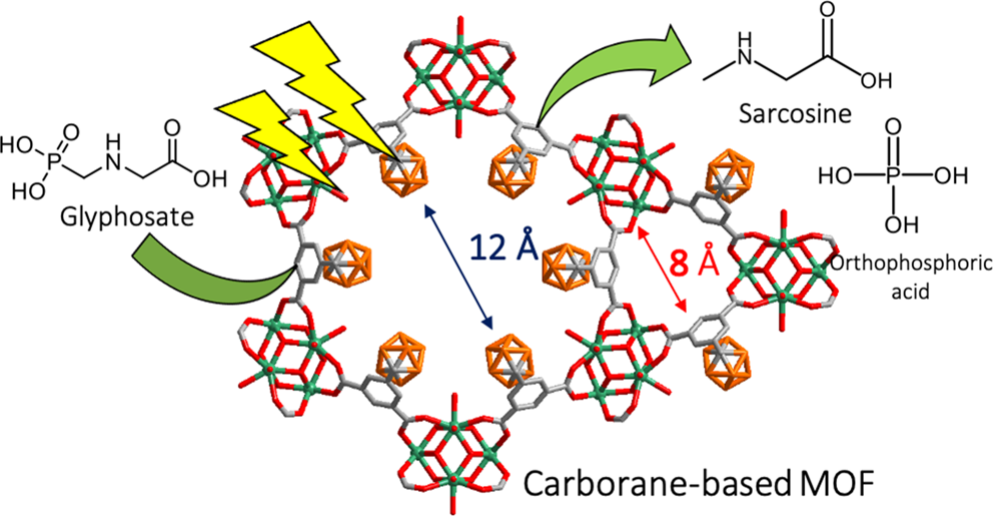

The removal of organophosphorus (OP) herbicides from water has been studied using adsorptive removal, chemical oxidation, electrooxidation, enzymatic degradation, and photodegradation. The OP herbicide glyphosate (GP) is one of the most used herbicides worldwide, leading to excess GP in wastewater and soil. GP is commonly broken down in environmental conditions to compounds such as aminomethylphosphonic acid (AMPA) or sarcosine, with AMPA having a longer half-life and similar toxicity to GP. Metal–organic frameworks (MOFs) are excellent materials for purifying OP herbicides from water due to their ability to combine adsorption and photoactivity within one material. Herein, we report the use of a robust Zr-based MOF with a *meta*-carborane carboxylate ligand (***m*CB-MOF-2**) to examine the adsorption and photodegradation of GP. The maximum adsorption capacity of ***m*CB-MOF-2** for GP was determined to be 11.4 mmol/g. Non-covalent intermolecular forces between the carborane-based ligand and GP within the micropores of ***m*CB-MOF-2** are thought to be responsible for strong binding affinity and capture of GP. After 24 h of irradiation with ultraviolet–visible (UV–vis) light, ***m*CB-MOF-2** selectively converts 69% of GP to sarcosine and orthophosphate, following the C–P lyase enzymatic pathway and biomimetically photodegrading GP. Circumventing the production of AMPA is desirable, as it has a longer half-life and similar toxicity to GP. The exceptional adsorption capacity of GP by ***m*CB-MOF-2** and its biomimetic photodegradation to non-toxic sarcosine make it a promising material for removing OP herbicides from water.

## Introduction

Organophosphorus compounds (OPs) are highly toxic synthetic compounds that include chemical warfare agents, pesticides, and herbicides.^1^ The high toxicity of OP-based chemical warfare agents and pesticides is attributed to their binding affinity to acetylcholinesterase, resulting in neuromuscular paralysis and death.^1−3^ Agriculturally, the less lethal but still toxic OP herbicides glyphosate (GP, Round-Up) and glufosinate (GF, Rely and Cheetah) work by inhibiting the shikimic acid pathway present in plants, preventing the biosynthesis of amino acids needed for growth.^4−6^ GP and GF herbicides are standard solutions used control destructive and invasive crop life, with GP being the most widely used herbicide in the U.S.^7,8^ However, their frequent use leads to undesirable amounts of residues in groundwater and food, posing a potentially serious risk to public health.^7^ Detection of GP in human urine samples is evidence of increased public exposure to OP-based herbicides, leading the Environmental Protection Agency (EPA) to set a maximum level for GP in wastewater at 0.7 g/L.^9,10^ Current water treatment standards employ various techniques to remove GP and its primary degradation product, aminomethylphosphonic acid (AMPA). AMPA has similar toxicity and longer half-life than its parent molecule and it can accumulate in the soil.^11−13^ AMPA is also problematic because it has been shown to interfere with DNA synthesis and repair in fish and amphibians and can have adverse effects on human blood cells.^14,15^ Therefore, developing effective technologies to remove toxic OP herbicides such as GP and GF and their metabolites (e.g., AMPA) from water is highly desirable.

To date, various methods for OP removal have been studied, including electrooxidation, adsorption, enzymatic biodegradation, and photocatalytic degradation.^16−28^ These methods possess several disadvantages, including their inability to capture OPs efficiently, high energy consumption, logistical difficulty, and except for enzymatic biodegradation, lack of control in the resultant products of OP degradation.^29^ Adsorption is a common strategy for GP removal due to its simple operation and low energy consumption, and adsorbents such as activated carbon (AC) can efficiently remove GP.^30^ On the other hand, research about GP degradation has focused on chemical oxidation via O_3_ or Cl_2_ or the traditional photocatalyst titanium dioxide (TiO_2_).^27^ Oxidative processes such as ozonolysis can degrade GP and AMPA, but residual O_3_ and Cl_2_ are often observed as byproducts.^28^ TiO_2_ can selectively degrade GP, but the major product often depends on the pH of the reaction solution, with sarcosine being favored at low pH.^31^ However, most studies report that both AMPA and sarcosine are produced with various catalysts, including TiO_2_, manganese dioxide (MnO_2_), and MnO_2_ minerals such as birnessite and ferrioxalate.^11,12,32−34^ Previously, work with TiO_2_ has shown that it can degrade up to 92% of GP,^33^ but the removal efficiency depends on many factors, including the reactor setup, lamp intensity, catalyst loading, pH, and GP concentration.^31,35^ Goethite or magnetite photocatalysts can also selectively degrade GP to sarcosine, but again the reaction must be buffered to pH 7.^36^ The above methods are reported as effective treatments for GP-contaminated water. Still, they lack selective interactions with the herbicide or require constant control over the pH of the reaction solution.^36,37^ Thus, it would be beneficial to develop a catalyst that can selectively degrade GP to avoid producing toxic AMPA without needing to control the pH.

**Figure 1:**
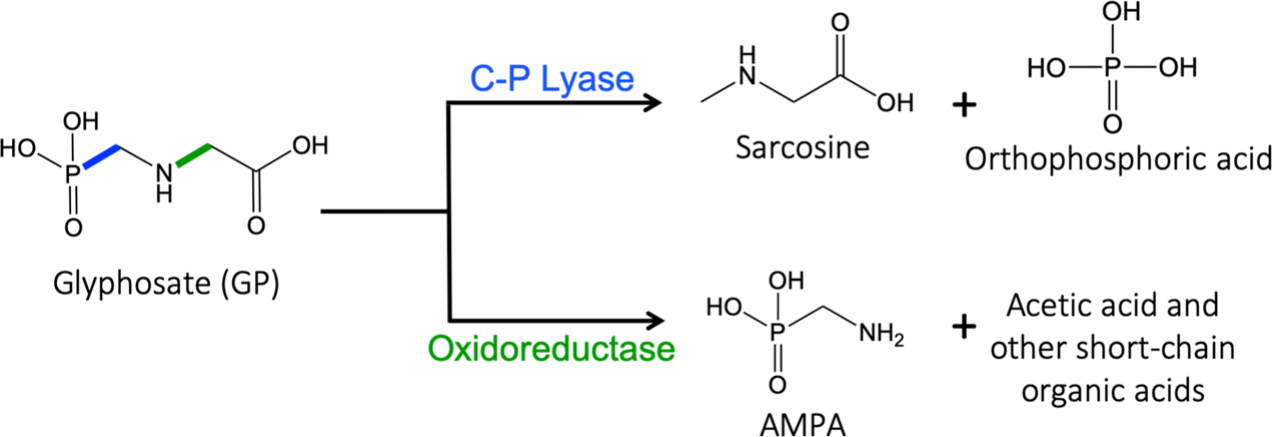
Schematic illustration of the photooxidation of GP under UV light in the presence of a catalyst. GP can be degraded to (i) sarcosine and orthophosphate or (ii) AMPA and short-chain organic acids depending on the enzymatic pathway (C–P lyase and oxidoreductase, respectively).

Currently, two known enzymes—C–P lyase and GP oxidoreductase (GOX)—can biodegrade GP (Figure 1). The enzymatic complex C–P lyase catalyzes the cleavage of the C–P bond, metabolizing GP to sarcosine (2-(methylamino)-acetic acid) and orthophosphate.^38^ Sarcosine produced through the C–P lyase pathway possesses no known toxicity and is metabolized to glycine by sarcosine oxidase.^37^ The GOX enzyme cleaves the C–N bond in GP, producing AMPA and glyoxylate or acetic acid.^32,33,37,38^ Unfortunately, the GOX biodegradation pathway often results in secondary contamination through the release of AMPA, which cannot be metabolized intracellularly.^37^ Therefore, the C–P lyase pathway is preferable as no toxic AMPA is produced.

Enzymatic biodegradation is highly selective, with selectivity attributable to the presence of a specific reaction center. Localization and capture of GP within a confined space could mimic enzyme selectivity, and the nanoconfined spaces within a porous material could allow for a greater degree of product selectivity. GP treatment technology requires improvement, as adsorption and degradation alone have yet to be demonstrated as efficient strategies for GP removal. Thus, developing superior methods to control GP pollution is central to public and environmental health. We envision that an ideal material for GP removal combines high adsorption and photodegradation capacities. This material should be water stable, photoactive, and porous, with localized and nanoconfined binding sites that allow for enhanced adsorption and selective GP photodegradation.

Metal–organic frameworks (MOFs) are ideal materials for combining adsorption and photoactivity. MOFs are a highly versatile class of porous materials made from metal clusters linked by organic ligands. To date, MOFs have shown great promise in many research areas,^39−46^ including in the efficient capture of herbicides^25,39,41,45,46^ and the photodegradation of organic pollutants such as dyes.^47^ Among these, zirconium (Zr)-carboxylate MOFs have received primary interest due to their chemical, thermal, and hydrolytic stabilities, mild synthetic conditions, and versatile connectivity.^44,48−52^ Zhu et al. first reported the adsorptive removal of OP herbicides GP and GF using UiO-67.^26^ They discovered that the Zr–O(H) and bridging Zr–O groups in the nodes of UiO-67 served as natural binding sites for the phosphonic group in the herbicides GP and GF.^26^ Zr-MOFs can also act as potent catalysts for the degradation of OP compounds due to the high concentration of Lewis acidic-Zr(IV) sites in the Zr_6_ clusters.^25^ Additionally, the steric and electronic microenvironments created in MOF pore spaces allow for reaction control.^53^ In this regard, the functionalized pore spaces of MOFs can mimic enzyme pockets, leading to increased reactivity and product selectivity.^54−56^ Thus, we envision that the high stability, adsorption capacity, and photocatalytic activity within the nanoconfined pore spaces of Zr(IV)-based MOFs make them excellent candidates for the adsorption and photodegradation of the OP herbicides GP and GF.

Icosahedral carboranes {1,*n*-C_2_B_10_H_12_ [*n* = 2 (*ortho*-), 7 (*meta*-) or 12 (*para*-)]} are a class of commercially available and exceptionally stable 3-dimensional-aromatic boron-rich clusters that possess material-favorable properties such as thermal and chemical stability and high hydrophobicity.^57−61^ The more electronegative carbon atoms contribute more electrons to cluster bonding than the boron atoms, which results in the carbon atoms effectively having an electron-withdrawing character.^62,63^*para*-Carborane has been used as a scaffold to grow MOF structures, resulting in the generation of highly stable and porous materials.^64−70^ We later developed a series of *ortho*-^71^ and *meta*-carborane^72−76^ based coordination polymers and MOFs, some with outstanding water stabilities. Herein, we describe the synthesis of a water-stable and porous Zr(IV)-carborane-based MOF with a tetracarboxylate *meta*-carborane ligand, denoted as ***m*CB-MOF-2**, for the capture and photodegradation of GP. We found that ***m*CB-MOF-2** has a larger adsorption capacity for both GP and GF compared to other adsorptive materials used for their removal to date. Density functional theory (DFT) calculations suggest that non-covalent intermolecular forces between the carborane-based linker and GP in the micropores of ***m*CB-MOF-2** are responsible for their enhanced interactions. Due to a higher observed uptake capacity of GP relative to GF by ***m*CB-MOF-2**, we investigated this material for the photodegradation of GP. We found that GP photodegradation by ***m*CB-MOF-2** is biomimetic, following the C–P lyase enzymatic pathway, forming only non-toxic sarcosine as a product without the requirement of pH control.

## Results and Discussion

### Synthesis and Characterization of ***m*CB-MOF-2**

**Figure 2:**
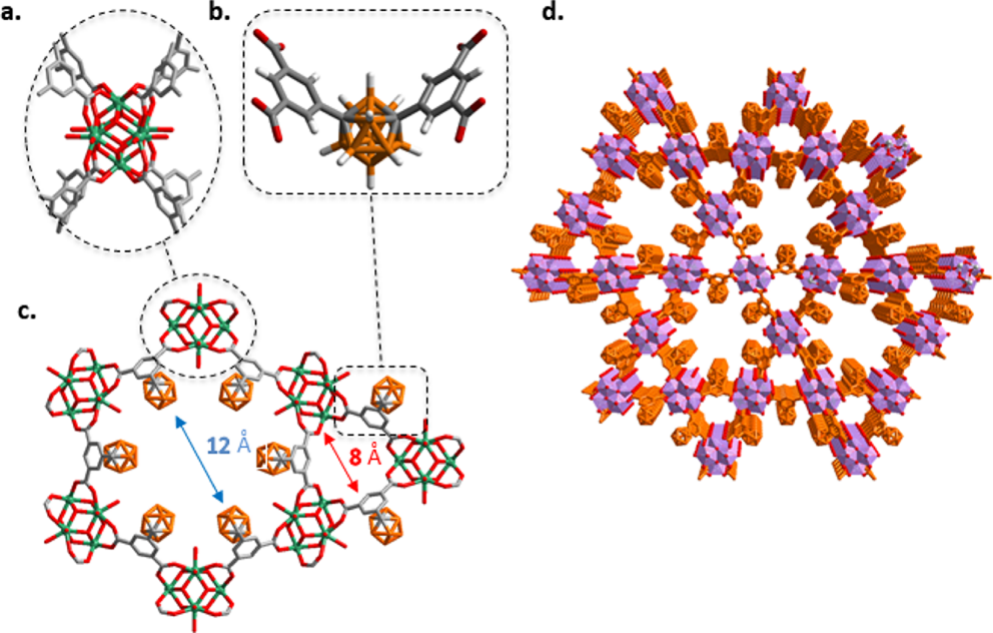
Crystal structure of ***m*CB-MOF-2**. (a) View of the 8-connected Zr_6_-cluster and (b) the carboxylate *m*CB-**L2** linker. (c) Axial cross-section view of the extended structures showing the 2-dimensional 4^4^ networks. (d) 3-Dimensional framework with hexagonal and triangular 1-dimensional channels, view of the “stacking” of (c). Structures to compose the porous network; Zr_6_ clusters are shown as violet polyhedra. Except in (b), H atoms are omitted for clarity. Color code: B, orange; C, gray; O, red; and Zr, green.

A new V-type (bent) bis-phenyl tetracarboxylic acid ligand derived from the *m*-carborane cluster [*m*CB-H_4_**L2**: 1,7-di(3,5-dicarboxyphenyl)-1,7-dicarba-*closo*-dodecaborane], was synthesized via Cu(I) coupling and oxidation.^77^ This new ligand, *m*CB-H_4_**L2**, was characterized by spectroscopic and analytical techniques (see the Supporting Information). Heating *m*CB-H_4_**L2** with ZrCl_4_ in the presence of formic acid and *N*,*N*-dimethylformamide (DMF) at 120 °C for 48 h yielded single, colorless prism-shaped crystals of ***m*CB-MOF-2** (Figure S1). Single-crystal X-ray diffraction (SCXRD) studies revealed that ***m*CB-MOF-2** crystallizes in the space group *P*6/*mmm* and possesses ***csq*** topology (Figure 2 and Table S1). Such a topology is commonly observed in Zr-MOF structures with planar tetracarboxylic ligands such as NU-1000, PCN-222, MOF-545, or MMMPF-6.^52^ The framework of ***m*CB-MOF-2** consists of octahedral Zr_6_-clusters linked by bent *m*CB-**L2** ligands (Figure 2). Each octahedral Zr_6_-unit is capped by μ_3_-O/OH groups providing a Zr_6_(μ_3_-O)_4_(μ_3_-OH)_4_ core. Eight octahedral edges of each Zr_6_O_4_(OH)_4_ core are connected to eight ***m*CB-MOF-2** units, with the remaining four Zr(IV) edges occupied by terminal OH/OH_2_ ligands.^78^ This gives rise to ***m*CB-MOF-2** with a molecular formula of [Zr_6_(μ_3_-O)_4_(μ_3_-OH)_4_(OH)_4_(H_2_O)_4_(*m*CB-**L2**)_2_] guest molecules, and it possesses hexagonal and triangular channels with pore diameters of 1.2 and 0.8 nm, respectively (Figure 2). The V-shape of our ligand imposes a shrink of the resultant network compared to ordinary planar tetracarboxylic linkers. The latter usually provides mesoporous materials,^51^ whereas our new material is microporous (Figures 2 and S2). After removing the guest solvent molecules, the total solvent-accessible volume of ***m*CB-MOF-2** was calculated to be 52.5% by PLATON.^79^ Combining the accessible volume and the density of the static structure of ***m*CB-MOF-2** gave a pore volume of 0.57 cm^3^/g.

**Figure 3:**
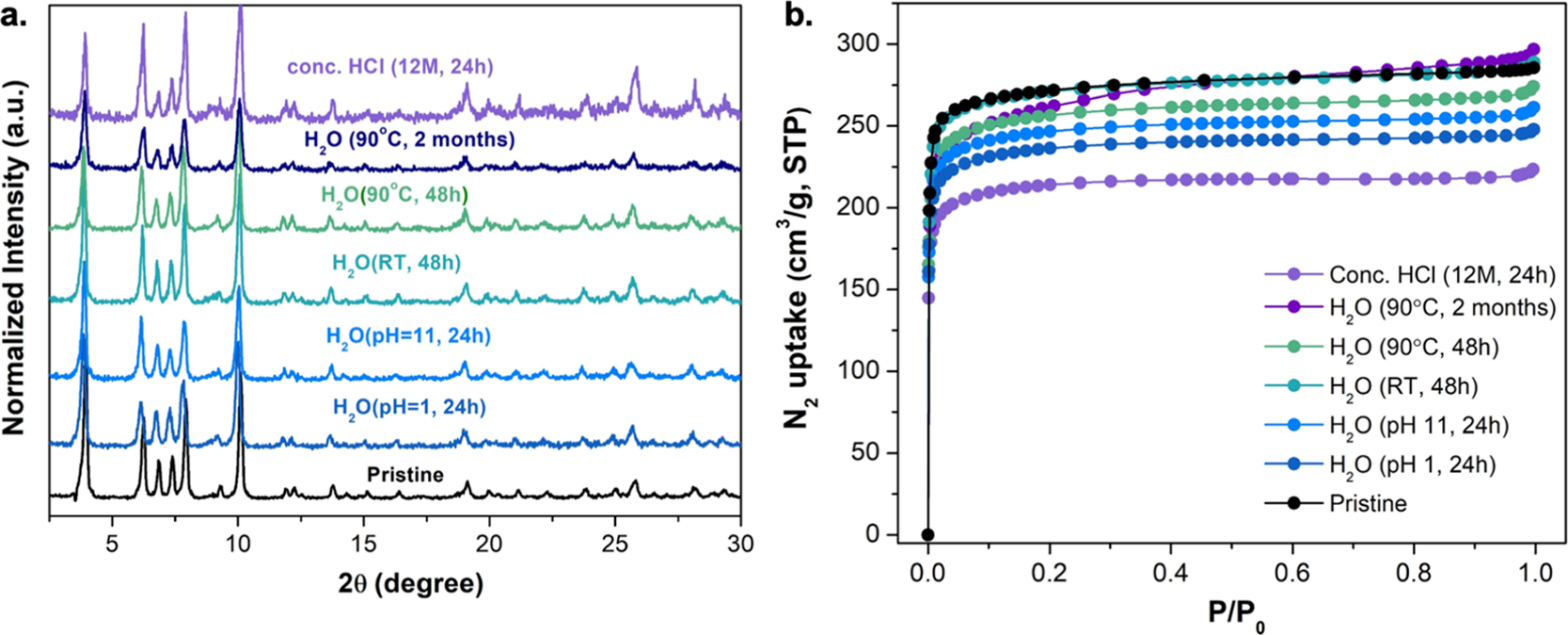
Comparison of (a) PXRD patterns and (b) N_2_ adsorption isotherms collected at 77 K and 1 bar for activated ***m*CB-MOF-2′** and after being treated under different conditions.

The bulk phase and analytical purity of ***m*CB-MOF-2** were confirmed by powder X-ray diffraction (PXRD, Figure S3), elemental analysis, and infrared spectroscopy (IR). IR spectra showed characteristic (Zr)O–H/O–H_2_ stretching bands (in the range 3600–3700 cm^–1^),^51,78,80^ and a B–H stretching band for the carborane fragments at 2606 cm^–1^ (Figure S4). Thermogravimetric analysis (TGA) of ***m*CB-MOF-2** after its immersion in acetone revealed that it is stable up to 270 °C (Figure S5). Variable temperature synchrotron wide angle X-ray scattering (WAXS) measurements showed that ***m*CB-MOF-2** retains its structural architecture up to 270 °C under dynamic vacuum, consistent with the TGA data (Figure S6). PXRD patterns indicated that the structure of the activated MOF, ***m*CB-MOF-2′**, is intact upon removal of the guest molecules from its cavities (Figure S3). Type I N_2_ isotherms collected at 77 K and 1 bar confirmed the microporous nature of ***m*CB-MOF-2′**, giving a Brunauer–Emmett–Teller (BET) surface area of 1095 m^2^/g (Figure 3b), and an experimental pore volume of 0.44 cm^3^/g, which is slightly lower than the calculated pore volume of the static structure (0.57 cm^3^/g). ***m*CB-MOF-2′** was also found to be porous to CO_2_ (273–313 K), CH_4_, and H_2_O vapors at 298 K, and H_2_ at 77 K and 1 bar (Figure S7).

Chemical stability was investigated by immersing ***m*CB-MOF-2′** in water under various conditions (RT, 90 °C, acidic or basic) for 24–48 h, followed by PXRD and BET (Figure 3a,b and Table S2). PXRD patterns of ***m*CB-MOF-2′** before and after incubation in a closed vial of water for 48 h at RT or 90 °C perfectly matched the simulated pattern derived from the single crystal structure of ***m*CB-MOF-2** (Figure 3a). Samples showed little change in their PXRD patterns after treatment under acidic (HCl, pH 1) or basic (NaOH, pH 11) conditions, in highly concentrated HCl (12 M) for 24 h, and even after being in water at 90 °C for 2 months (Figure 3a). While PXRD analysis showed no change in crystallinity, porosity investigations revealed minor changes in the N_2_ uptake (77 K and 1 bar) and BET surface areas under those harsh conditions (Figure 3b and Table S2). We also evaluated the possible influence of the carborane units on the hydrophobic properties of ***m*CB-MOF-2′**. Contact angle (Θ_c_ ∼ 0°) measurements indicated that the surface of ***m*CB-MOF-2′** is hydrophilic, and water adsorption isotherms of ***m*CB-MOF-2′** collected at 298 K (Figure S7d) showed a two-step process, which can be correlated to the filling of the two cavities present in the structure. The overall data demonstrate the excellent chemical and hydrolytic stability of ***m*CB-MOF-2**, in line with the presence of Zr_6_-clusters and carborane units in the MOF structure.^44,71,72,81^

### Adsorption of Glyphosate and Glufosinate

**Figure 4:**
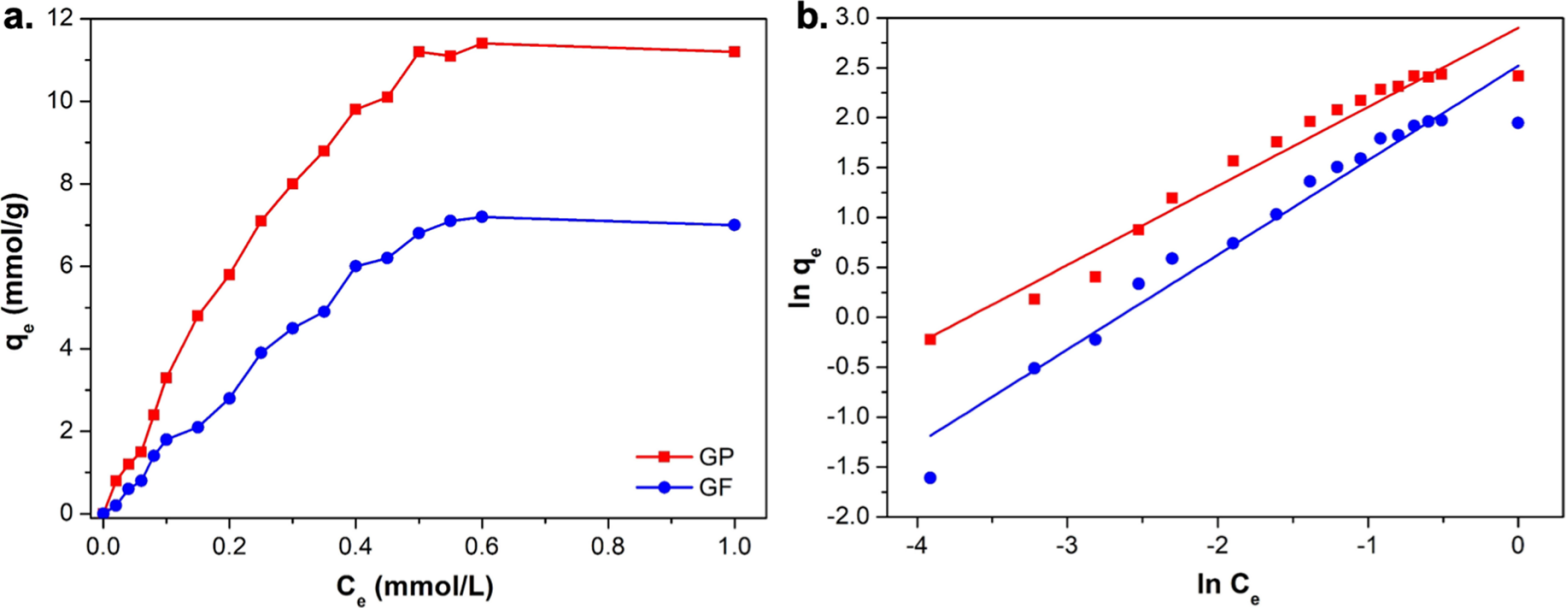
(a) Solution adsorption isotherms of GP and GF using ***m*CB-MOF-2′** at 295 K and (b) Freundlich model plots.

Due to the stability of ***m*CB-MOF-2** in aqueous, acidic, and basic solutions, we explored the capture of two of the most frequently used OPs in agriculture, GP and GF. We first tested the capture of GP and GF with ***m*CB-MOF-2′** at room temperature (295 K) for 48 h (Figure 4a). The isotherms revealed the relationship between the system’s equilibrium concentration (*C*_e_) and the amount of herbicide adsorbed (*q*_e_) on the MOF. As, to the best of our knowledge, only a few reports have been published on the adsorption of these herbicides on MOFs, we evaluated the experimental isotherms using the Langmuir and Freundlich isothermal models to understand the adsorption behavior thoroughly.^25,26,82^

**Figure 5:**
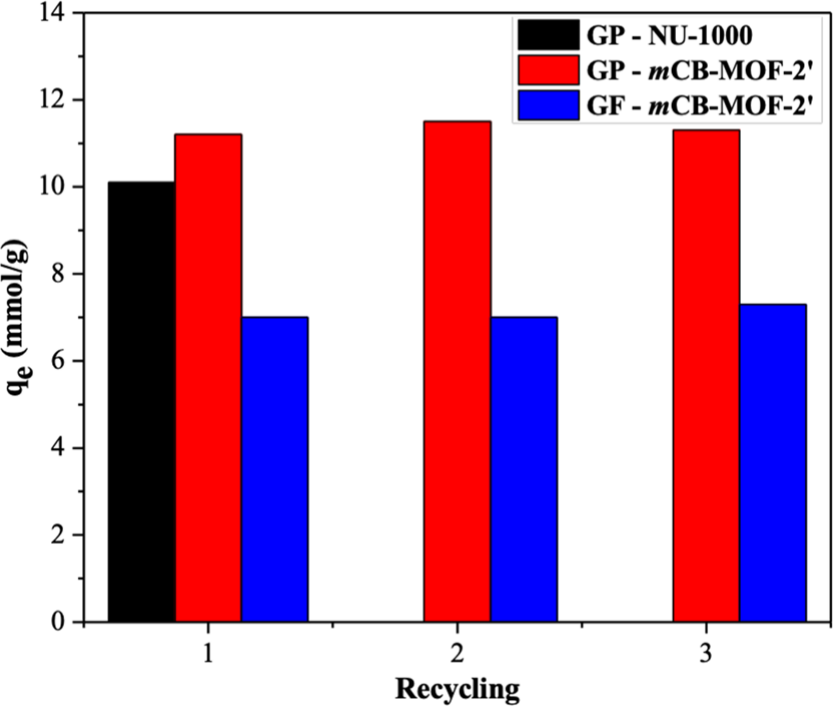
Regeneration of ***m*CB-MOF-2′** and cycling of GP and GF adsorption after 48 h. For comparison, the adsorption of GP on NU-1000 was used (1 h, after which the uptake was saturated).

Analysis of each model showed that the empirical Freundlich model (Figure 4b) has a better fit than the Langmuir model (Figure S8 and Table S3) in both cases, contrary to the adsorption on UiO-67 or NU-1000.^25,26,82^ Other isothermal models, such as Temkin and Dubinin–Radushkevich models, do not provide a better fit to the experimental isotherms (Figures S9 and S10 and Table S4). Our data suggest that the adsorption of GP and GF on ***m*CB-MOF-2′** follows the Freundlich isothermal model, indicating the energetical equivalency and heterogeneity of the adsorption sites. Both *n* values were less than 1.0 (Table S3), demonstrating favorable adsorption of GP and GF on ***m*CB-MOF-2′**. The maximum GP and GF adsorption capacities (*q*_max_) on ***m*CB-MOF-2′** were 11.4 and 7.2 mmol g^–1^, respectively. The higher adsorption value for GP indicates that ***m*CB-MOF-2′** presents distinctive affinities for OPs with differing molecular structures. Previous work has shown that the Lewis acidic Zr_6_-clusters have a high affinity for the Lewis basic phosphonate functional groups of GP and GF, and therefore, we expect the Zr metal nodes in the framework to be the primary adsorption sites for the herbicides.^25,83^ Thus, the presence of a methyl group on the phosphonate moiety of GF (not present in GP) might diminish its bonding with the Zr–OH groups in ***m*CB-MOF-2′**, as observed in the case of UiO-67.^26^ Nevertheless, the adsorption capacities of GP and GF on ***m*CB-MOF-2′** are higher than NU-1000 (8.97^25^ or 10.1 mmol g^–1^ in the present work), UiO-67 (7.90 mmol g^–1^ for GP),^25^ and other reported materials to date (Table S5). We further evaluated the stability and recyclability of ***m*CB-MOF-2′** following the adsorption of GP and GF, with PXRD patterns showing that ***m*CB-MOF-2′** retains its structure after adsorption in 0.05 mmol L^–1^ GP or GF solution for 48 h (Figures S11 and S12). ***m*CB-MOF-2** was then washed with acidified water three times to remove any GP or GF from the pores, activated at 60 °C for 4 h, and the regenerated material was reused for the GP and GF uptake experiments. No significant decrease in the BET surface area of the material (Figures S13 and S14) nor in the adsorption capacity for GP and GF after at least three adsorption cycles (Figure 5) was observed, demonstrating that ***m*CB-MOF-2′** could be recycled and reused for GP and GF capture. Comparing the adsorption capacities of GP and GF on different porous materials (Table S5) reveals that ***m*CB-MOF-2′** is an excellent candidate for the adsorptive removal of OPs for environmental pollution management.

### Density Functional Theory of GP Adsorption by ***m*CB-MOF-2′**

**Figure 6:**
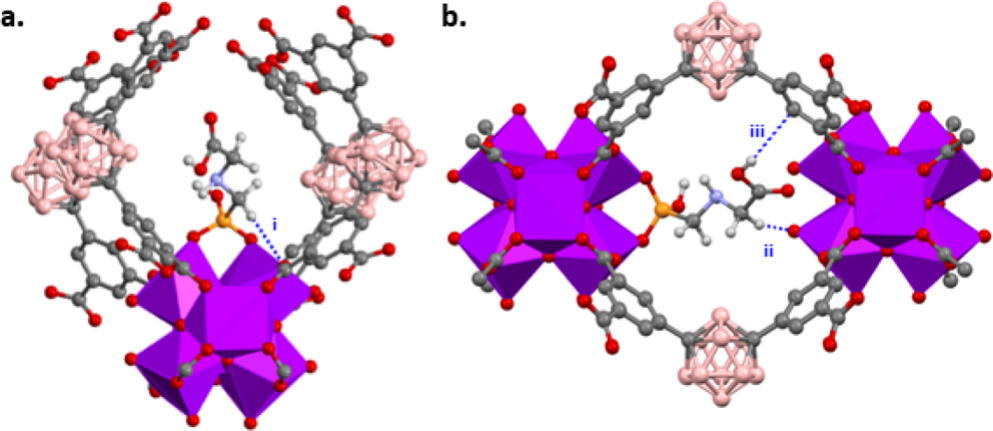
DFT calculated conformations of GP binding in ***m*CB-MOF-2′**. (a) GP in the triangular pores of ***m*CB-MOF-2′** shows a non-covalent interaction with the surrounding framework [blue dotted line (C–H···O, (i): H···O 2.855 Å, CHO 134°)] and (b) in the c-pores of ***m*CB-MOF-2′** showing non-covalent interactions with the surrounding framework {blue dotted lines [C–H···O, (ii): H···O 2.196 Å; CHO 162°; O–H···C, and (iii): H···C 2.884 Å, OHC 126°]}.

DFT calculations suggest that the adsorption conformations of a GP molecule in ***m*CB-MOF-2′** involve two different adsorption sites: (i) in the triangular channels (Figure 6a) and (ii) between two adjacent Zr nodes in orthogonal windows to the hexagonal and triangular channels (Figure 6b), named c-pores as in NU-1000.^84^ According to the DFT calculations, GP coordinates to ***m*CB-MOF-2′** through the phosphonic acid end of the molecule and also shows hydrogen bonding between GP and the surrounding framework in both pores (triangular and c-pores; Figures 6 and S15). The related DFT calculations for the mesoporous NU-1000 showed the GP molecules adsorb in the c-pores and mesoporous hexagonal channels through the phosphonic acid, but hydrogen bonding with the surrounding framework is only observed in the microporous c-pores.^84^ A binding energy of −241 kJ/mol was found for the c-pores of ***m*CB-MOF-2′** (Figure 6b) and −201 kJ/mol for NU-1000. It is thought that the smaller c-pores’ size in ***m*CB-MOF-2′** compared to NU-1000 may explain the higher binding energy for our MOF. For the other adsorption site, the narrow triangular channels in ***m*CB-MOF-2′** (Figure 6a) provide the possibility of non-covalent intermolecular forces between the carborane-based linker and GP molecule, which is not observed in the mesoporous hexagonal channels in NU-1000. Thus, a much higher binding energy was calculated for these adsorption sites in ***m*CB-MOF-2′** (−231.0 kJ/mol) than for the mesopores in NU-1000 (−192.9 kJ/mol). The DFT calculations demonstrate the strong interaction between GP and our microporous carborane-based framework, combined with higher Zr_6_ cluster density (0.57 mmol/g in ***m*CB-MOF-2′** and 0.46 mmol/g in NU-1000), explaining the higher GP uptake compared to mesoporous NU-1000.

### Optoelectronic Properties of ***m*CB-MOF-2′** and NU-1000

**Figure 7:**
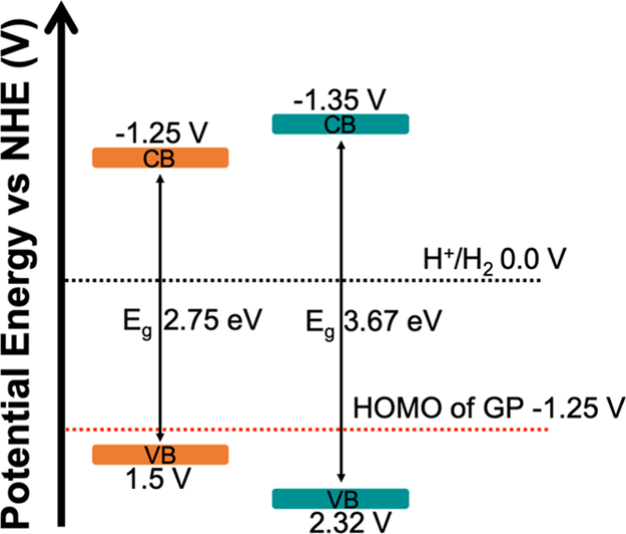
Schematic energy level diagrams of NU-1000 (orange) and ***m*CB-MOF-2** (cyan).

To investigate the optoelectronic properties of ***m*CB-MOF-2′** and, for comparison, NU-1000, we studied the UV–vis diffuse reflectance spectra (DRS) of these two MOFs (Figure S12a). ***m*CB-MOF-2** and NU-1000 samples exhibit an absorption band edge at approximately 330 and 470 nm (Figure S16a), corresponding to band gaps (*E*_g_) of 3.67 and 2.75 eV, respectively, according to the Tauc plots (Figure S16b). Compared to ***m*CB-MOF-2**, a wider spectral absorption band and narrower band gap were observed in NU-1000. The conduction band (CB) and valence band (VB) positions of ***m*CB-MOF-2** and NU-1000 were determined based on Mott–Schottky analyses (Figure S16c,d). The positive slopes of Mott–Schottky plots indicated the typical n-type semiconductors for both MOFs. Notably, the smaller slope for ***m*CB-MOF-2** showed higher donor density than for NU-1000. The flat potentials determined from the intersection value were calculated to be −1.55 and −1.45 eV versus Ag/AgCl for ***m*CB-MOF-2** and NU-1000, respectively. Usually, the bottom of the CB is approximated by the flat band potential; thus, the CB edges are −1.35 eV for ***m*CB-MOF-2** and −1.25 eV for NU-1000. The more negative CB edge of ***m*CB-MOF-2** suggests a stronger photo-reducing ability than NU-1000. From the band gap energies and CB positions of ***m*CB-MOF-2** and NU-1000, the VB edges were calculated to be 2.32 eV for ***m*CB-MOF-2** and 1.5 eV for NU-1000. The more positive VB for ***m*CB-MOF-2** indicates a stronger GP oxidative ability than NU-1000 (Figure 7). As the highest occupied molecular orbital (HOMO) of GP lies at −1.25 V versus the normal hydrogen electrode (NHE),^85^***m*CB-MOF-2** has a stronger thermodynamic driving force for GP oxidative degradation compared to NU-1000.

### Photodegradation of Glyphosate

**Figure 8:**
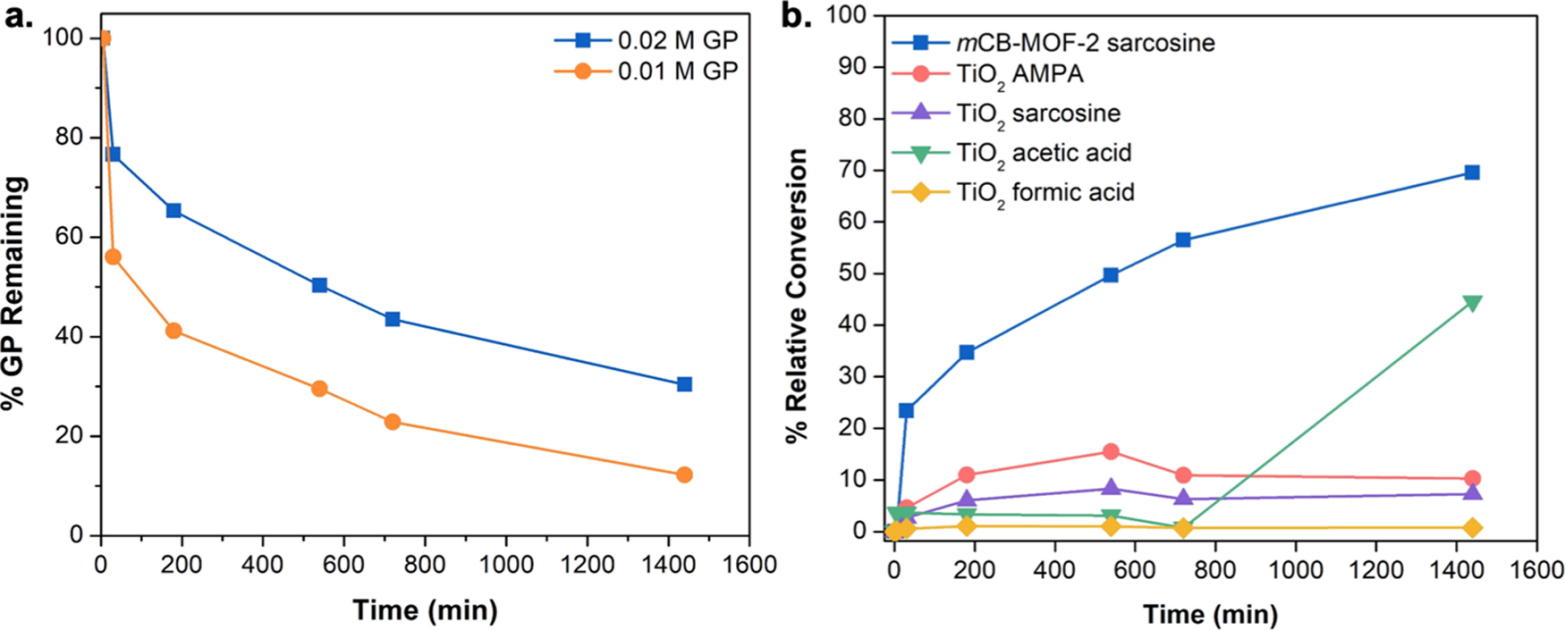
(a) Photodegradation results show that in 24 h, ***m*CB-MOF-2** degrades 69 and 87% of 0.02 and 0.01 M GP to sarcosine, respectively; and (b) conversion of 0.02 M GP solution to sarcosine and AMPA using ***m*CB-MOF-2** or TiO_2_. In 24 h, TiO_2_ degrades 63% of 0.02 M GP, non-selectively, into a mixture of sarcosine, AMPA, acetic, and formic acid.

Considering the optoelectronic properties, exceptional stability, large GP adsorption capacity, and potential photoactivity (Figure S16) of ***m*CB-MOF-2**, the photodegradation of GP was investigated. The photodegradation efficiency of ***m*CB-MOF-2** was evaluated using 0.02 and 0.01 M GP solutions (Figure 8a). Interestingly, characterization of the resulting products with ^1^H and ^31^P NMR indicated that ***m*CB-MOF-2** selectively decomposes GP to sarcosine and orthophosphate by following the C–P lyase enzymatic pathway (Figures 2, S17a, and S18a). At the same time, irradiation of the 0.02 M GP solution without a photocatalyst showed no GP degradation (Figure S19). After irradiation for 24 h, ***m*CB-MOF-2** degraded 69 and 87% of GP when 0.02 and 0.01 M solutions were used, respectively, with sarcosine and orthophosphate being the only products after photodegradation (Figures 8a, S17a, and S18a). Gas chromatography–mass spectrometry (GCMS) also confirmed the selective degradation of GP by ***m*CB-MOF-2** (Figures S20–S25 and Table S7). PXRD collected post-irradiation verified that ***m*CB-MOF-2** is stable under UV light (Figure S26), and N_2_ isotherms showed a slight decrease in the BET surface area from 1095 to 995 m^2^/g (Table S2).

To better understand the efficiency of ***m*CB-MOF-2** in the photodegradation of GP, the same experiments were performed using the common photocatalyst TiO_2_. The latter produces five times less sarcosine than ***m*CB-MOF-2** after a 30 min irradiation (Figure 8b). Contrary to ***m*CB-MOF-2**, ^1^H and ^31^P NMR analysis indicated that both AMPA and sarcosine are produced with TiO_2_ in addition to acetic and formic acid (Figures S17b and S18b). While TiO_2_ can still photodegrade GP, it is non-selective and produces the toxic and longer-lived product AMPA. ***m*CB-MOF-2** demonstrates a greater efficiency for converting GP to sarcosine than the TiO_2_ standard. We suspect that the carborane-decorated, nanoconfined pores and high adsorption capacity of ***m*CB-MOF-2′** increase its photodegradation efficiency and selectivity, as GP can access additional active sites within the MOF pore, and reactions are not limited to the surface.

**Figure 9:**
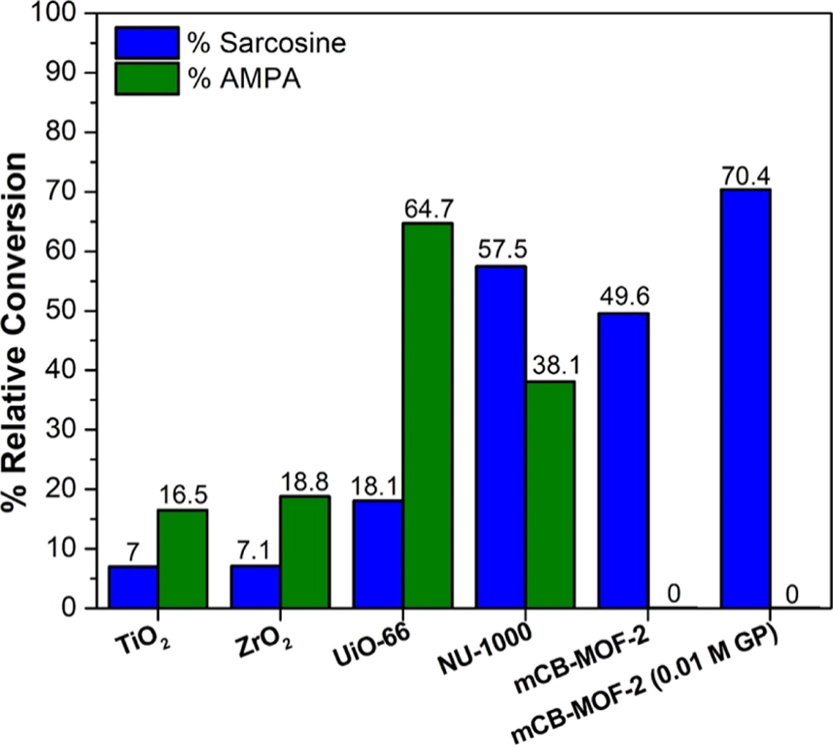
Comparing photocatalysts in the degradation of 0.02 M GP solution after 9 h. ***m*CB-MOF-2** is the only material among them that is selective, degrading GP into sarcosine only. All other materials degrade GP into sarcosine (blue) and AMPA (green). GP photodegradation to sarcosine with ***m*CB-MOF-2** increases using a lower (0.01 M) concentration.

Intrigued by the selectivity of ***m*CB-MOF-2** in GP photodegradation, we performed a 9 h photodegradation experiment using non-porous TiO_2_ or zirconium dioxide (ZrO_2_) and porous MOFs that have previously been studied for GP adsorption (UiO-66 and NU-1000) (Figure S27). Consistent with the above results, TiO_2_ was non-selective and less efficient for the degradation of GP (Figure 9) compared to the MOF photocatalysts. ZrO_2_ was investigated to probe the impact of the Zr–O(H) sites during photodegradation. ZrO_2_ also performed relatively poorly for GP degradation and produced both AMPA and sarcosine (Figure 9). The poor performances of TiO_2_ and ZrO_2_ could be due to their reduced porosity, as any photodegradation reactions would be limited to the surface.

On the other hand, GP photodegradation with porous UiO-66 was slightly more efficient than ***m*CB-MOF-2** (Figures 9 and S27). However, it also lacked selectivity as AMPA was the primary product (64.7%), compared to sarcosine (18.1%). Among all the tested photocatalysts, mesoporous NU-1000 showed the best performance for GP photodegradation (Figure 9). While NU-1000 was the most efficient, it was also non-selective, producing both AMPA (38.1%) and sarcosine (57.5%). The wider spectral absorption band and narrower band gap observed in NU-1000, compared with those for ***m*CB-MOF-2** (Figure S16), signify its larger light utilization. The latter agrees with the higher GP degradation for NU-1000. However, the more positive VB for ***m*CB-MOF-2** (Figure 7) indicates a stronger thermodynamic driving force for GP oxidation than NU-1000. The stronger photo-redox ability of ***m*CB-MOF-2**, in addition to the nanoconfinement of GP in the micropores of our MOF, could explain the preferential C–P bond activation by ***m*CB-MOF-2**.^38^

**Figure 10:**
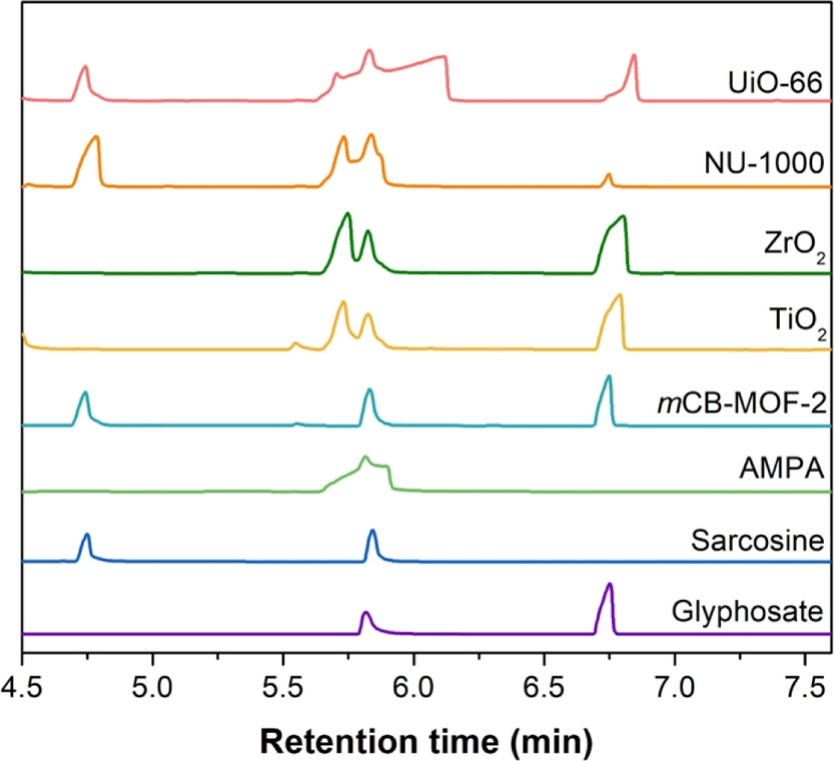
GCMS TIC for standards and tested photocatalysts after 9 h GP irradiation. AMPA (*t*_R_ 5.79 min) co-elutes with a byproduct of the derivatization process, most likely dimethyl glutarate (*t*_R_ 5.82 min). GCMS detected no AMPA for ***m*CB-MOF-2**, but it has a distinctive peak and mass spectrum for the other photocatalysts.

We analyzed each photocatalyst’s 9 h sample by GCMS to ensure selectivity and confirm the degradation products. Due to the low volatility and high polarity of GP and AMPA, derivatization was necessary for GCMS analysis of the samples and standards (see the Supporting Information).^86^ The GCMS total ion chromatogram (TIC) (Figure 10) shows the retention time (*t*_R_) for the standards and samples. As shown in Figure 10, AMPA (*t*_R_ 5.79 min) co-elutes with dimethyl glutarate (DMG, *t*_R_ 5.82 min, 91% match from MS database), likely a byproduct of the derivatization reaction, as it was also present in the derivatized standards. Notably, the TIC shows that the AMPA and DMG peaks are unresolved for the AMPA standard and all photocatalysts except for ***m*CB-MOF-2**, where no peak for AMPA is observed (Figures 10, S20, and S21). Additionally, MS results for ***m*CB-MOF-2** around the retention time of AMPA showed no major ions indicative of derivatized AMPA. In contrast, the other photocatalysts had distinct ions (*m*/*z*) for AMPA in their mass spectra (Figures S21–S25). Thus, the GCMS results support our conclusion from the ^1^H and ^31^P NMR, as no AMPA was observed in the ***m*CB-MOF-2** sample for either technique and provides additional evidence for the selective GP degradation route.

We tested the uptake of AMPA by ***m*CB-MOF-2** (following methods from the GP and GF uptake experiments) and found that after 1 h, ***m*CB-MOF-2** captured 86.8% of the AMPA (200 ppm) present in the solution, compared to 93.2 and 14.6% for NU-1000 and TiO_2_, respectively (Table S8 and Figure S28). This result was not surprising, as AMPA is structurally similar to GP, and the low uptake for TiO_2_ can be attributed to its lack of porosity. Upon capture, the AMPA-loaded ***m*CB-MOF-2** was irradiated with light. The BET surface area of ***m*CB-MOF-2** after photocatalysis was found to be 916 m^2^/g, slightly higher than the surface area of the AMPA-loaded ***m*CB-MOF-2** before irradiation (807 m^2^/g, Figure S29). No degradation products were observed by ^1^H NMR after photocatalysis; however, traces of orthophosphate could be detected by ^31^P NMR (Figure S30). These data suggest that although photodegradation of AMPA is possible, it seems negligible under the present reaction conditions.

The above experiments indicate that AMPA might be sequestered in ***m*CB-MOF-2** and therefore prevented its detection in the supernatant after GP degradation. We note however that if a significant amount of AMPA was produced during GP photodegradation and subsequently absorbed and blocked the active sites of ***m*CB-MOF-2**, the GP degradation rate would have greatly decreased. This is not the case and sarcosine production only increased over the 24 h reaction (Figure 8b). These results indicate that even in the event that ***m*CB-MOF-2** adsorbs AMPA and could potentially photodegrade it, the GP degradation process into sarcosine is much faster.

### Photodegradation Pathway

The carborane moieties within the porous framework of ***m*CB-MOF-2** are expected to contribute significantly toward the high GP uptake observed. Carboranes are strong electron-withdrawing groups.^57,62^ Due to this, the carboxylic acid functional groups on the *m*CB-**L2** ligands become more acidic, weakening their conjugate bases. As a result, the carboxylate groups may coordinate less strongly to each Zr(IV), causing Zr to be less electronically satisfied and, therefore, more Lewis acidic. Thus, the Zr(IV) clusters of ***m*CB-MOF-2** may experience an electron deficiency, enhancing their electrophilic behavior.^80^ This more significant degree of electron-receptivity is proposed to activate the Zr(IV) sites and promote increased capture through coordination with the Lewis basic phosphonic group in GP. It is important to note that the adsorption site (Zr(IV) clusters) and pore environment in ***m*CB-MOF-2** are effectively surrounded by carborane fragments (Figures 6 and S15), so that adsorbed GP molecules are nanoconfined in the narrow pores of the MOF.

**Scheme 1:**
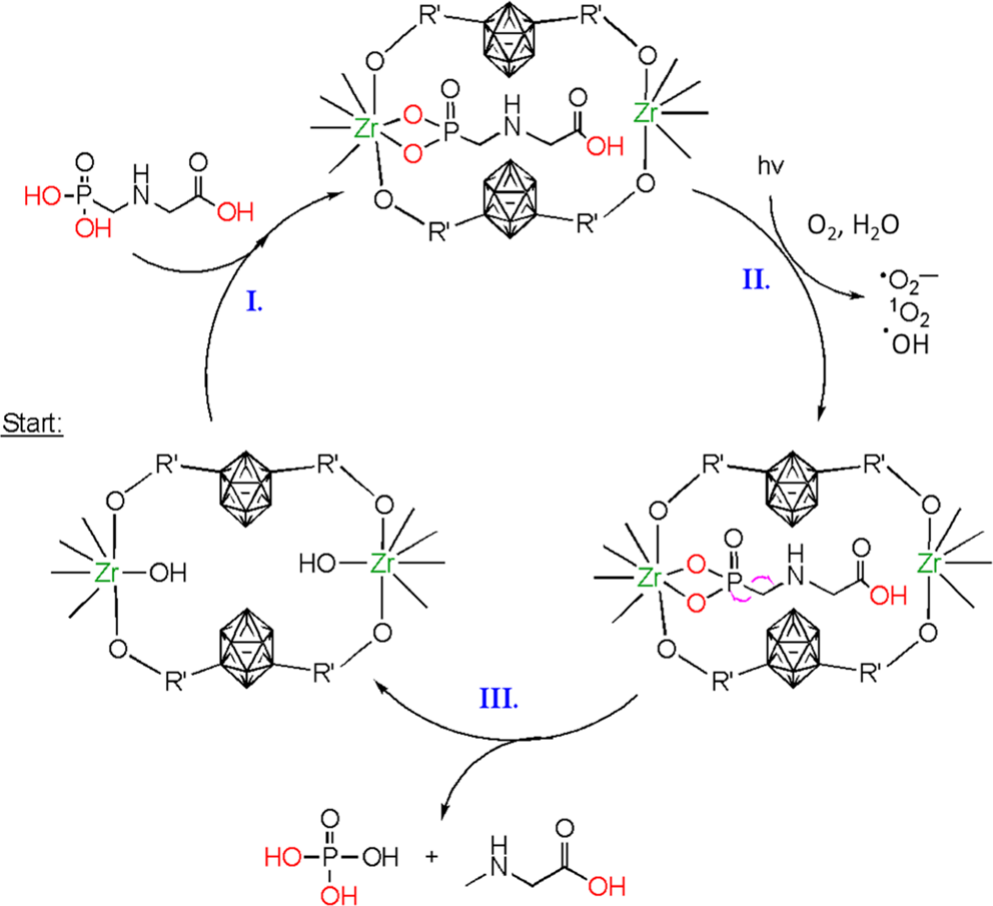
GP Photodegradation Cycle Starting from an accessible pore of ***m*CB-MOF-2**: (I) GP adsorption within the pore; (II) UV irradiation and photogeneration of reactive oxidation species; and (III) C–P lyase biomimetic photodegradation of GP into sarcosine and phosphoric acid.

Irradiation of a photocatalyst produces both electrons (e^–^) and holes (h^+^). The holes can directly oxidize pollutants or oxidize oxygen to produce reactive oxygen species (ROS). ROS are generally accepted as the primary oxidizing agents in photodegradation reactions and can include hydroxyl radicals (^•^OH), superoxide radicals, and singlet oxygen (^1^O_2_).^87−90^ Electron spin resonance (ESR) spectra were recorded to investigate the generation of ROS (^•^OH, ^•^O_2_^–^, and ^1^O_2_) in our photocatalytic experiments. We have confirmed the presence of reactive species ^1^O_2_, as well as and ^•^OH, by conducting ESR measurements on our MOF in the presence of (2,2,6,6-tetramethylpiperidin-1-yl)oxyl (TEMPO) and 5,5-dimethyl-1-pyrroline N-oxide (DMPO), respectively. No TEMPO–^1^O_2_, DMPO–^•^OH signals were observed in the absence of UV light (Figures S32–S35). Upon irradiation, a 1:1:1 signal corresponding to TEMPO–^1^O_2_, a 1:1:1:1 signal corresponding to DMPO–, and a 1:2:2:1 signal corresponding to DMPO–^•^OH appeared, suggesting the generation of ^1^O_2_, ^•^O_2_^–^, and ^•^OH, respectively.^91^ Both ^•^OH and ^•^O_2_^–^ can act as nucleophiles to attack the electrophilic P-atom of GP (step III in Scheme 1), cleaving the C–P bond. The ESR data confirm the generation of ROS but do not confirm the dominance of a specific ROS in the photodegradation of GP. To evaluate the role of each ROS in the observed photodegradation reaction, we have further investigated the effect of radical scavengers. Sodium azide (NaN_3_), triethylamine (TEA), triethanolamine (TEOA), and *tert*-butanol (BuOH) are commonly employed as radical scavengers for ^1^O_2_,^92^^•^O_2_^–^, h^+^, and ^•^OH, respectively.^47,93^ To investigate the reactive species responsible for the photodegradation of GP by ***m*CB-MOF-2**, the aforementioned ROS scavengers were added to the photocatalytic reaction setup. The results show that NaN_3_ is the more efficient scavenger, although all scavengers significantly inhibited the degradation of GP (Figure S31). In the presence of NaN_3_, the degradation of GP at 30 min and 9 h was 0 and 6.21%, respectively. In comparison, without NaN_3_, 43.96 and 70.46% of GP were degraded in the same time intervals (0.02 M GP solution). Even though the scavenger experiments suggest that ^1^O_2_ has a dominant role in the photodegradation of GP, all ROS contribute to this catalytic reaction.

Based on the DFT calculations, ESR experiments, and ROS trapping, the photocatalytic degradation mechanism of GP by ***m*CB-MOF-2′** is proposed in Scheme 1. As previously stated, the electron-withdrawing carborane moieties within the porous framework of ***m*CB-MOF-2** cause Zr(IV) clusters to be more Lewis acidic, and coordination of Zr(IV) sites with the Lewis basic phosphonic group in GP is expected to activate the C–P bond. When ***m*CB-MOF-2** is irradiated by UV–vis light, it generates photoexcited electrons that transition to the CB, leaving holes in the VB. The photogenerated electrons in the CB can reduce dissolved or adsorbed O_2_ to produce ^•^O_2_^–^, which can then be oxidized to ^1^O_2_ by photogenerated holes in the VB (step II in Scheme 1). As a powerful oxidant, ^1^O_2_ could oxidize GP into non-toxic sarcosine and orthophosphate (step III in Scheme 1). Concurrently, holes in the VB can oxidize water to produce ^•^OH (step II in Scheme 1). Photogenerated ^•^OH and ^•^O_2_^–^ can participate in nucleophilic attack on the P-atom via an S_N_2-like substitution. The O-atom of the incoming nucleophile is incorporated into the phosphate to form the observed orthophosphate, and the carbon-centered radical hydrolyzes an H_2_O molecule to form sarcosine. Thus, our carborane-decorated MOF is highly selective in GP photodegradation compared to the other photocatalysts employed in this work, producing sarcosine and orthophosphate exclusively.

Previous studies on GP photodegradation using manganese oxide have proposed a charge transfer mechanism where the metal site is reduced, either from an electron transfer from GP, creating a phosphate-based radical,^94^ or electron transfer from water resulting in metal reduction and water oxidation to create radicals that subsequently attack the weakened C–P bond.^12^ Carbon-centered radical mechanisms have also been proposed in the photodegradation of GP.^12,31,94^ Nucleophiles present in the reaction pot (H_2_O, −OH, and ^•^OH) can participate in nucleophilic attack of the P-atom in GP, resulting in the incorporation of an oxygen atom from the nucleophile to form orthophosphate. Oxygen incorporation by the phosphate group has previously been documented through isotope (^18^O) analysis, and the O-atom likely comes from dissolved O_2_ or H_2_O,^12,95^ which were abundant in our reaction system. Since the reaction pH was between 2 and 4, protons and water were also available. Therefore, the carbon-centered radical could acquire a proton from the solution or hydrolyze an H_2_O molecule to form sarcosine.^12,94^

The observed selectivity on GP degradation by our carborane ***m*CB-MOF-2** framework can be explained by a combination of factors, including (i) a more Lewis acid character of the Zr metal centers, as consequence of the weaker coordination with the carborane-based carboxylate linkers and (ii) the nanoconfinement of the absorbed GP in the nanopores of ***m*CB-MOF-2**. The coordination of GP through the phosphonic acid to the Zr metal center is expected to further activate the C–P bond and make it more susceptible to nucleophilic attack by ^•^O_2_^–^ and ^•^OH, as well as to oxidation by ^1^O_2_. It is well known that the reactivity and selectivity of reactive species such as ROS can be greatly enhanced when conducted in confined nanospaces.^96−98^ The GP degradation process by ***m*CB-MOF-2** is taking place in the nanoconfined pores of the MOF, and this is thought to facilitate the observed selectivity.

## Conclusions

We report, for the first time, the use of a thermal and hydrolytically stable MOF made of Zr(IV) and a tetracarboxylate carborane ligand, namely, ***m*CB-MOF-2**, for the adsorption of GP and GF and biomimetic photodegradation of GP. These herbicides are of great concern owing to their indiscriminate use and negative impacts on human and environmental health. The microporous nature of the carborane-decorated ***m*CB-MOF-2** leads to the efficient capture of both GP and GF. The synergy between the Zr(IV) node and carborane linker of ***m*CB-MOF-2** is vital for the efficient degradation of GP. ***m*CB-MOF-2** selectively degrades GP into sarcosine and orthophosphate, acting in a biomimetic fashion by following the C–P lyase pathway. Our MOFs’ selective degradation to non-toxic sarcosine is significant, as it does not require control over the pH of the reaction solution. This is compared to traditionally accepted TiO_2_, which produces undesired and toxic AMPA and non-toxic sarcosine. Band structure analysis of ***m*CB-MOF-2** revealed our MOF as an n-type semiconductor with a high donor density and low CB edge, explaining the strong photo-redox ability of our material. The observed selectivity on GP degradation by ***m*CB-MOF-2** is thought to be due to the enhanced Lewis acid character of the Zr metal centers, binding of GP, and its nanoconfinement into the carborane-decorated channels of the MOF. Our findings highlight the potential for ***m*CB-MOF-2** to be used in herbicide capture and degradation to non-hazardous products. We envision the employment of this MOF in a continuous process in agricultural settings. The unique biomimetic photodegradation of GP by ***m*CB-MOF-2** provides an opportunity for future investigations into the mechanisms of GP photodegradation and how this can be expanded to other herbicides.

## Supporting Information Available

Syntheses, experimental details, WAXS spectra, PXRD patterns, isotherms, ^1^H and ^31^P NMR spectra, and GCMS chromatograms (PDF)
