# Supplementary material for: Biomimetic Photodegradation of Glyphosate in Carborane-Functionalized Nanoconfined Spaces
Source: J Am Chem Soc. 2023 Jun 20;145(25):13730–41. doi: 10.1021/jacs.3c02019 (PMC10311523; doi:10.1021/jacs.3c02019)
Supplement: Supplementary file 1 — ja3c02019_si_001.pdf [file ja3c02019_si_001.pdf]

Supporting Information

Biomimetic Photodegradation of Glyphosate in Carborane-functionalized Nanoconfined Spaces

Lei Gan,[a][b] Makenzie T. Nord,[c] Jacob M. Lessard,[c] Noah Q. Tufts,[c] Arunraj Chidambaram,[d][e] Mark E. Light,[f] Hongliang Huang,[g] Eduardo Solano,[h] Julio Fraile,[a] Fabián Suárez-García,[i] Clara Viñas,[a] Francesc Teixidor,[a], Kyriakos C. Stylianou,*[c] José G. Planas*[a]

[a] Institut de Ciència de Materials de Barcelona (ICMAB-CSIC), Bellaterra, Spain.

E-mail: jginerplanas@icmab.es

[b] Institute of Physical and Theoretical Chemistry, Graz University of Technology, 8010 Graz, Austria.

[c] Materials Discovery Laboratory (MaD Lab), Department of Chemistry, Oregon State University, 153 Gilbert Hall, OR 97331, Corvallis, Oregon, USA.

E-mail: kyriakos.stylianou@oregonstate.edu

[d] Institute of Chemical Sciences and Engineering, École Polytechnique Fedérale de Lausanne (EPFL Valais), Rue de l'Industrie 17, 1951 Sion, Switzerland.

[e] Chemspeed Technologies AG, Wölferstrasse 8, 4414 Füllinsdorf, Switzerland

[f] Department of Chemistry, University of Southampton, Highfield, Southampton SO17 1BJ, UK.

[g] State Key Laboratory of Separation Membranes and Membrane Processes, School of Chemistry and Chemical Engineering, Tiangong University, Tianjin 300387, China.

[h] NCD-SWEET beamline, ALBA Synchrotron Light Source, 08290 Cerdanyola del Vallès, Barcelona, Spain.

[i] Departament of Material Chemistry, Instituto de Ciencia y Tecnología del Carbono, INCAR-CSIC, Oviedo, Spain.

Materials.

All chemicals were commercially available and used as received. All synthetic procedures were carried out in air unless noted otherwise.

Characterization and methods.

Attenuated Total Reflection Fourier Transformed Infrared (ATR-FTIR) spectra were recorded using a PerkinElmer Spectrum One spectrometer equipped with a Universal ATR sampling accessory. Spectra were collected with 2 cm^{-1} spectral resolution in the 4000-650 cm^{-1} range.

The precursor (*m*CB-**L1**) to the ligand and the ligand itself (*m*CB-**L2**) were characterized by **^{1}H, ^{13}C, and ^{11}B Nuclear Magnetic Resonance (NMR)** spectra using a Bruker Advance-400 spectrometer. Spectra were collected in deuterated dimethylsulfoxide (*d*-DMSO), unless noted, and referenced to the residual solvent peak for ^{1}H and ^{13}C NMR or to $BF_3{\cdot}OEt_2$ as an external standard for ^{11}B NMR. Chemical shifts are reported in ppm and coupling constants in Hertz. Characterization of the products of glyphosate photodegradation was done in deuterium hydroxide (D_2O) using a 500 MHz Bruker Ascend (BBO probe) or a 700 MHz Bruker Ultrashield Plus (cryoprobe) NMR spectrometer. Multiplet nomenclature is as follows: s, singlet; d, doublet; t, triplet; br, broad; m, multiplet.

Gas Chromatography-Mass Spectrometry (GCMS) using an Agilent 7820A GC paired with a 5975 series MSD was also used to characterize the photodegradation products. The GC was operated in splitless mode using an HP-5MS column and helium as the carrier gas (0.7 mL/min). The injection port was 270°C (1 µL injection volume) with a 4.40-minute solvent delay. The column was held at 70°C for 2 minutes, then ramped 30°C/min to 170°C for 1 minute and 75°C

/min to 270°C for two minutes for a total run time of 9.67 minutes. The MS was operated in electron impact (EI) mode with source and quad temperatures of 230°C and 150°C, respectively.

Elemental analyses were obtained using a Thermo (Carlo Erba) Flash 2000 Elemental Analyser, configured for wt.%CHN.

Thermogravimetric Analysis (TGA) was performed on an nSTA 449 F1 Jupiter-Simultaneous TGA-DSC or SDT Q600 V8.3 Build 101 instrument under an N_2 atmosphere (heating rate: 5 ^{o}C/min; temperature range: 25 ^{o}C to 600 ^{o}C).

Gas sorption-desorption of CO_2 at 273-313 K, N_2 at 77 and 313 K, H_2 at 77K, CH_4, and H_2O at 298 K measurements were performed using IGA001, ASAP2020, and Belsorp Max II surface area analyzer, respectively. The sample was first degassed at 130 °C for 12 h.

Crystals for **X-ray Diffraction (XRD)** were prepared under inert conditions immersed in perfluoropolyether or paratone as protecting oil for manipulation. Suitable crystals were mounted on MiTeGen Micromounts™ and used for data collection at BL13 (XALOC)[1] at the ALBA synchrotron (Spain) with an undulator source and channel-cut Si(111) monochromator and Kirkpatrick-Baez focusing mirrors with a selected wavelength of 0.72932 Å. An MD2M-Maatel diffractometer fitted with a Dectris Pilatus 6M detector was employed. The sample was kept at 100 K with an Oxford Cryosystems 700 series Cryostream. The structure was solved using the ShelXT 2014/5 (Sheldrick, 2014) structure solution program using the direct phasing methods solution method and by using Olex2 as the graphical interface.[2] The model was refined with version 2016/6 of ShelXL using Least Squares minimization.[3] Highly disordered solvent, identified as six ethanol per formula unit, was treated using a solvent mask (Squeeze). A summary of crystal data is reported in Table S1. Powder X-ray Diffraction (PXRD) was recorded at room

temperature on a Siemens D-5000 diffractometer with Cu Kα radiation (λ = 1.54056 Å, 45 kV, 35 mA, increment=0.02º).

Morphological features were first examined by optical microscopy and then by **Scanning Electron Microscopy (SEM)** with a QUANTA FEI 200 FEGESEM microscope.

Water contact angles were measured using a Krüss DSA 100 device at room temperature using water as the probe fluid (9 μL).

Inductively Coupled Plasma – Mass Spectrometry (ICP-MS) measurements were carried out on an Agilent ICP-MS 7700x instrument. ICP-OES measurements were also collected on a Horiba JY Ultrex instrument.

Wide Angle X-ray Scattering (WAXS) patterns were recorded on the NCD-SWEET beamline at the ALBA synchrotron light source (Spain). An X-ray beam of 8 keV (λ = 1.54 Å) was set using a Si (111) channel-cut monochromator. The scattered radiation was recorded using a Rayonix LX-255HS area detector. The sample-to-detector distance and the reciprocal space calibration were obtained using Cr_2O_3 as a standard calibrant. The MOF was introduced in a borosilicate capillary and heated to 300 ºC using a Linkam TMS-350 capillary stage (10 ºC/min from 25 ºC; resting 30 minutes after every 50 ºC increase) under dynamic vacuum. Data was reduced from 2D images to 1D profiles via azimuthal integration using PyFAI.[4]

Electron Spin Resonance (ESR)

Electron spin resonance (ESR) signals of the radicals trapped by (2,2,6,6−tetramethylpiperidin-1-yl)oxyl (TEMPO) and 5,5-dimethyl-1-pyrroline *N*-oxide (DMPO) were detected using a JEOL JES-FA200 electron paramagnetic resonance spectrometer at room temperature at X-band frequencies (v ≈ 9.2 GHz)

Synthesis of 1,7-di(3,5-dimethylphenyl)-1,7-dicarba-*closo*-dodecaborane (*m*CB-L1).

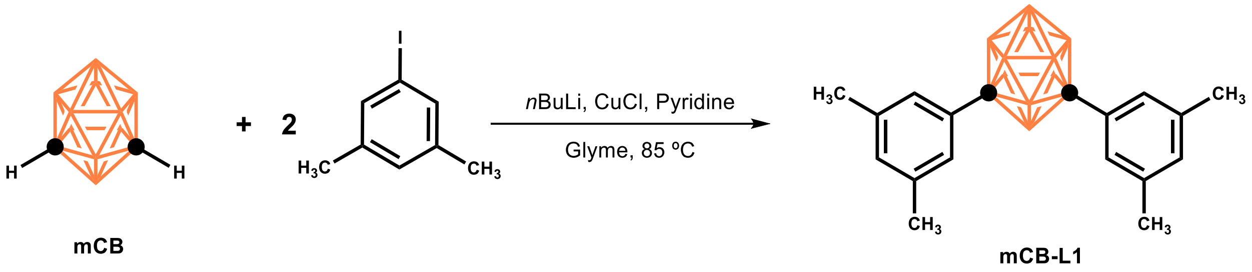

The synthesis of the 1,7-di(3,5-dicarboxyphenyl)-1,7-dicarba-closo-dodecaborane ligand (mCB-H_4L_2) was adapted from the literature procedure.[5] The experiment was conducted under a nitrogen atmosphere in a round-bottomed flask equipped with a magnetic stir bar. 1.00 g (6.93 mmol) of *m*-carborane (mCB) was added to an oven-dried Schlenk flask. The flask was evacuated and backfilled with N_2 three times, then 1,2-dimethoxyethane (50 mL) was added to the flask. Once the mCB was totally dissolved, 10.2 mL (1.6 M in hexane, 16.32 mmol) of *n*-BuLi was added dropwise at 0ºC. The mixture was then stirred at room temperature for 20 min, and then CuCl (2.38 g, 24.04 mmol) was added to the solution. The mixture was stirred for 20 min, and then 1.11 mL (0.010 mmol) of pyridine and 2.01 mL (13.86 mmol) of 5-I-*m*-xylene were added. The solution was heated and refluxed at 85ºC until the TLC showed the original compound was almost completely consumed. The cooled mixture was diluted with 200 mL ether and allowed to stand for 2 h. The precipitate was filtered off, and the solution was extracted three times with an HCl (3 M) solution. The diethyl ether was removed by rotatory evaporation, providing a sticky solid that was filtered through a silica gel column (ethyl acetate: petroleum ether = 1:10). The filtrate was concentrated using a rotary evaporator to obtain **mCB-L1** as a white solid (1.54 g; 63.04%).

$^{1}H\{^{11}B\}$ NMR (400 MHz, $CDCl_3$): δ 2.32 (s, 6H, CH_3), δ 2.61 (br, 5H, BH), δ 6.94 (s, 2H, C_6H_3), δ 7.09 (s, 4H, C_6H_3); $^{11}B\{^{1}H\}$ NMR (400 MHz, $CDCl_3$): δ -6.10 (s, 2B), δ -10.73 (s, 5B), δ -13.27 (s, 2B), δ -15.26 (s, 1B). IR (ATR; selected bands; cm^{-1}): ν 3061 (CH); 2915 (CH); 2599 (BH).

Synthesis of 1,7-di(3,5-dicarboxyphenyl)-1,7-dicarba-closo-dodecaborane (*m*CB-H_4L2).

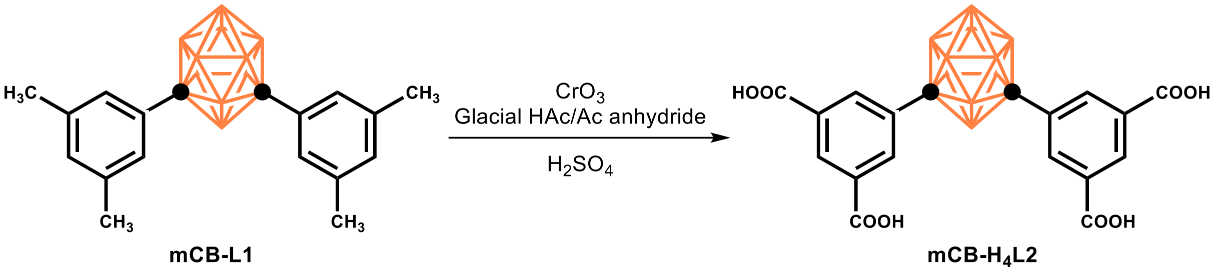

The procedure was adapted from a literature procedure.[5] 6.93 g (69.3 mmol) of CrO_3 was added in small portions to a stirred mixture of 1.54 g (4.37 mmol) of **mCB-L1**, 60 mL glacial acetic acid, 30 mL acetic anhydride, and 6.23 mL of concentrated H_2SO_4. The dark green mixture was stirred at room temperature for 2 hours and then poured into 100 mL of distilled water. A precipitate was filtered off and washed with distilled water to remove the green chromium residues. The off-white solid was recrystallized by dissolving it in a Na_2CO_3 solution, filtering it, and then acidifying it with an HCl (12M) aqueous solution. The white precipitate that appeared was filtered off to obtain 1.89 g (57.6%) of pure **mCB-H_4L2**.

$^{1}H\{^{11}B\}$ NMR (400 MHz, DMSO-d_6): δ 2.52 (br, 10H, B-H), δ 8.45 (s, 2H, C_6H_3), δ 13.66 (br s, 4H, COOH); $^{11}B\{^{1}H\}$ NMR (400 MHz, DMSO-d_6): δ -8.51 (br, 10B); IR (ATR; selected bands; cm^{-1}): ν 3081 br (OH, CH); 2607 (BH); 1693 (C=O from carboxylate). Elemental analysis (%) calculated for $C_{18}H_{20}B_{10}O_8$: C 45.76, H 4.27; Found: C 45.03, H 4.85.

Synthesis of [$Zr_6(\mu_3\text{-}O)_4(OH)_4(OH)_4(H_2O)_4$(mCB-$L_2)_2$] (***m*CB-MOF-2**).

*m*CB-H_4L_2 (20 mg, 0.0423 mmol) and $ZrCl_4$ (29 mg, 0.1269 mmol) were dissolved in DMF (5 mL). Formic acid (2.0 mL) was added to this solution in an 8-dram vial. The vial was closed and heated at 120 °C in an oven for 48 h, followed by slow-cooling to room temperature for 10 h. Colorless crystals of ***m*CB-MOF-2** were collected and washed with DMF (25 mg, yield 52.6 %). IR (ATR; selected bands; cm-1): 2606 (BH); 1656 (C=O from carboxylate); 3600-3700 (-OH/H_2O

from Zr cluster). Elemental analysis (%) calculated for $Zr_6C_{54}B_{20}H_{96}O_{41}N_6$: C 28.84, H 4.30, N 3.74; Found: C 28.64, H 4.03, N 3.89. As synthesized, ***m*CB-MOF-2** crystals were immersed in acetone (20 mL) and replaced once a day for three days, filtered, and dried in air. The latter was then activated by heating at 130 ºC under dynamic ultrahigh vacuum for 12h to form ***m*CB-MOF-2'**.

Herbicide and AMPA adsorption experiments.

An aqueous stock solution of GP, GF, or AMPA (200 ppm) was prepared by dissolving GP ($C_3H_8NO_5P$, MW: 169.07) or GF ($C_5H_{12}NO_4P$, MW: 181.1) in deionized water. GP or GF solutions with different concentrations of 0.01-0.5 mmol L^{-1} (0.01, 0.03, 0.05, 0.1, 0.15, 0.2, 0.3, 0.4, and 0.5 mM) were prepared by diluting the stock solution with water. The GP or GF concentrations were determined by measuring phosphorus using ICP-OES. Adsorption experiments were conducted at 25 °C, using 10 mg of ***m*CB-MOF-2'** in 10 mL of GP or GF solution. The solution was allowed to stir for 48 h to determine the adsorption capacity of the MOF. After centrifugation, the amount of adsorbed GP or GF was measured from the difference between the initial (C_0) and equilibrium (Ce) concentrations in the supernatant. The equilibrium uptake was calculated by the equation:

$$q_e = \frac{V(C_0 - C_e)}{W}$$

Where q_e (mmol g^{-1}) is the equilibrium adsorption capacity of GP or GF on ***m*CB-MOF-2'**, V is the volume of OP solution (L), and W is the weight of the used adsorbents (g).

The adsorption isotherms for GP and GF using both the Langmuir and Freundlich models on ***m*CB-MOF-2'** are provided in Figures S8 and 4b, respectively. The obtained parameters and the correlation coefficients (R^2) are presented in Table S3. The results showed that the empirical Freundlich model has a better fit than the Langmuir model in both cases, contrary to the adsorptions on UiO-67 or NU-1000.[6]

The Langmuir isothermal model is based on the assumption of monolayer adsorption, in which the adsorbate only combines with a finite number of open, active sites that are identical and equivalent.[82] However, the empirical Freundlich isothermal model describes non-ideal and

reversible adsorption, which can be applied to multilayer adsorption without restrictions on monolayer formation.[83] Langmuir adsorption was modeled with the following mathematical expression:

$$\frac{C_e}{q_e} = \frac{1}{K_L\, q_m} + \frac{C_e}{q_m}$$

where C_e is the equilibrium concentration of OP herbicide (mmol L^{-1}), q_e is the amount of OP adsorbed at equilibrium (mmol g^{-1}), K_L represents the Langmuir constant (L $mmol^{-1}$) that relates the adsorption energy and affinity of binding sites, and q_m denotes the maximum adsorption capacity (mmol g^{-1}). Freundlich adsorption mathematical expression is as follows:

$$\ln q_e = \ln K_F + n \ln C_e$$

where K_F ($mmol^{1-n}$ L^n g^{-1}) represents the Freundlich constant, which is related to the adsorption capacity of the adsorbent, and *n* is a parameter that indicates the adsorption intensity. The value of *n* reflects the type of isotherm to be favorable ($0 < n < 1$), irreversible ($n = 0$), or unfavorable ($n > 1$).

The adsorption data were also studied using the Temkin and Dubinin-Radushkevich adsorption models (Figures S9, S10 and Table S4) by using the following equations:

Temkin equation: $q_e = B \ln A + B \ln C_e \; (B = \frac{RT}{b})$

Dubinin-Radushkevich equation: $\ln q_e = \ln q_m - \beta\varepsilon^2 \; (\varepsilon = RT ln(1 + \frac{1}{Ce}))$

Where B, the constant related to heat of sorption(J/mol); A, the Temkin isotherm equilibrium binding constant (L/mol); b, the Temkin isotherm constant; R, the gas constant (8.314J/mol/K); T, the absolute temperature; q_m, the theoretical saturation capacity (mmol/g); β, a constant related to the mean free energy of adsorption per mole of the adsorbate (mol^2/kJ^2); ε, the Polanyi potential.

As implied in the equations, the parameters were determined by plotting the $\mathbf{q_e}$ *vs* $\mathbf{\ln C_e}$ in Temkin model and $\mathbf{\ln q_e}$ *vs* $\boldsymbol{\varepsilon^2}$ in Dubinin-Radushkevich model, then the correlation coefficient $\boldsymbol{R^2}$ were also obtained.

AMPA adsorption studies were performed by immersing 10 mg of ***m*CB-MOF-2'** in 200 ppm AMPA solution. The uptake of AMPA was investigated at different times using ICP-OES monitoring the concentration of phosphorus in the solution.

Computational Studies.

DFT calculations were carried out using the CP2K code.[7] All calculations employed mixed Gaussian and planewave basis sets. Core electrons were represented with norm-conserving Goedecker-Teter-Hutter pseudopotentials.[8-10] The valence electron wavefunction was expanded in a double-zeta basis set with polarization functions,[11] along with an auxiliary plane wave basis set with an energy cutoff of 400 eV. The generalized gradient approximation exchange-correlation functional of Perdew, Burke, and Enzerhof (PBE)[12] was used. Each configuration was optimized with the Broyden-Fletcher-Goldfarb-Shanno (BGFS) algorithm with SCF convergence criteria of 1.0×10^{-8} au. To compensate for the long-range van der Waals dispersion interaction between the adsorbate and the ***m*CB-MOF-2'**, the DFT-D3 scheme [13] with an empirical damped potential term was added into the energies obtained from exchange-correlation functional in all calculations.

Photodegradation studies.

All photodegradation experiments were conducted using a 300 W Newport xenon arc lamp utilizing a Newport-Oriel Instruments OPS-A500 power supply. Irradiation was conducted using UV-Vis light (200-800 nm). All irradiations took place with stirring of 1000 rpm throughout their entirety. A typical photodegradation solution was made by adding 2.0 mL of 0.02 M GP solution to 50 mg of MOF in a 1-dram vial (0.0287 M ***m*CB-MOF-2**), which was placed at a fixed distance from the xenon lamp for the assigned irradiation time. For TiO_2, the photodegradation solutions were made using 6.9 mg of TiO_2 in 3.0 mL of 0.02 M GP solution (0.0287 M TiO_2). The photodegradation experiments of GP using ZrO_2 (6.9 mg), NU-1000 (62.7 mg), and UiO-66 (42.9 mg) were conducted by adding 2.0 mL of 0.02 M GP solution to 1-dram vials equipped with stir bars.

The 0.02 and 0.01 M GP solutions using ***m*CB-MOF-2** or TiO_2 were irradiated for 5, 30, 180, 540, 720, and 1440 minutes. The 0.02 M GP solutions used for the comparisons with ZrO_2, NU-1000,

and UiO-66 were irradiated for 540 minutes (9 hours). Sodium azide (NaN_3), triethylamine (TEA), triethanolamine (TEOA), and *tert*-butanol (BuOH) experiments were done using roughly 0.012 mM of each scavenger, the same amount of ***m*CB-MOF-2** (50 mg) and GP solution concentration (2.0 mL of 0.02 M GP). For the AMPA photocatalysis experiments, 50 mg of ***m*CB-MOF-2** was immersed in an AMPA (0.02 M) solution and irradiated for 60 minutes.

Characterization of photodegradation products by ^{1}H NMR.

Sodium 3-(trimethylsilyl)-1-propane sulfonate (DSS) was used as a reference material. For each NMR sample, 75 µL of 1.01 mM DSS was used. First, ^{1}H NMR was obtained for GP (δ (ppm) 3.92 (s) and δ 3.26, 3.24 (d)), aminomethylphosphonic acid (AMPA) (δ (ppm) 3.09, 3.07 (d)), and sarcosine (δ (ppm) 2.72 (s) and δ 3.60 (s)) standards. ^{1}H NMR analysis was conducted on the UV-Vis-irradiated GP sample solutions. ^{31}P NMR (proton-decoupled) spectra were obtained for GP (δ (ppm) 8.71 (s)), AMPA (δ (ppm) 10.82 (s)), sarcosine (no ^{31}P peak expected or observed), and the 1440-minute (24 h) irradiation sample for ***m*CB-MOF-2** and TiO_2. The strong water signal observed in the ^{1}H NMR spectra of the photodegradation samples (δ (ppm) 4.7 (s)) was suppressed using a solvent suppression pulse program with excitation sculpting, a gradient and perfect echo from the Bruker library (zgesgppe).

Each ^{1}H NMR peak was integrated after normalizing the reference peak (DSS) to the number of protons giving rise to the signal at 0 ppm for DSS. The relative conversion of GP to sarcosine and AMPA was calculated using the formula:

$$M_x = \left(\frac{I_x}{I_{DSS}}\right)\left(\frac{N_{DSS}}{N_x}\right)(M_{DSS})$$

where M_x is the molarity (mol/L) of glyphosate, sarcosine, or AMPA, M_{DSS} is the molarity of the DSS reference solution (1.01 x 10^{-4} M), I_x is the sum of the integration values for the observed ^{1}H NMR peak for either sarcosine or AMPA, N_{DSS} is the number of nuclei giving rise to the DSS peak ($N_{DSS} = 9$), and N_x corresponds to the number of nuclei giving rise to the glyphosate ($N_x = 4$), sarcosine ($N_x = 5$), and AMPA ($N_x = 2$), signals.

The relative percent conversion was found by taking the ratio of the concentration found above for one compound over the total calculated concentration. For example, the percent conversion to sarcosine for ***m*CB-MOF-2** was found using:

$$\%\ conversion_{\boldsymbol{sarcosine}} = \left[\frac{M_{sarcosine}}{(M_{GP} + M_{AMPA} + M_{DSS} + M_{sarcosine})}\right] \times 100\%$$

GCMS Derivatization Procedure and Characterization of Photodegradation.

Derivatization of standards (glyphosate, AMPA, and sarcosine, 25 – 200 ppm) and samples (9 h ***m*CB-MOF-2**, NU-1000, UiO-66, TiO_2, and ZrO_2) was carried out according to a literature procedure with slight modifications.[14] Briefly, 1 mL of standard or sample solution in DI H_2O was evaporated from the 1-dram vial and dissolved in 1 mL of H_2O:MeOH:HCl (160:40:2.7), which was evaporated on a hot plate. Then 800 µL of trifluoroacetic anhydride (TFAA) and 400 µL trifluoroethanol (TFE) were added and placed in a 105°C sand bath for 1 h. The standards and samples were then cooled to room temperature and left on a hot plate (~60°C) to evaporate TFAA and TFE. The resulting residue was redissolved in ethyl acetate for GCMS analysis (1 µL injection). Table S6 shows the major ions observed for the standards, which are consistent with the literature,[14] in addition to a byproduct of the derivatization process (dimethyl glutarate) at 5.82 min. Figures S16-S21 show the total ion chromatograms and mass spectra obtained for the derivatized standards and samples.

Figures and Tables

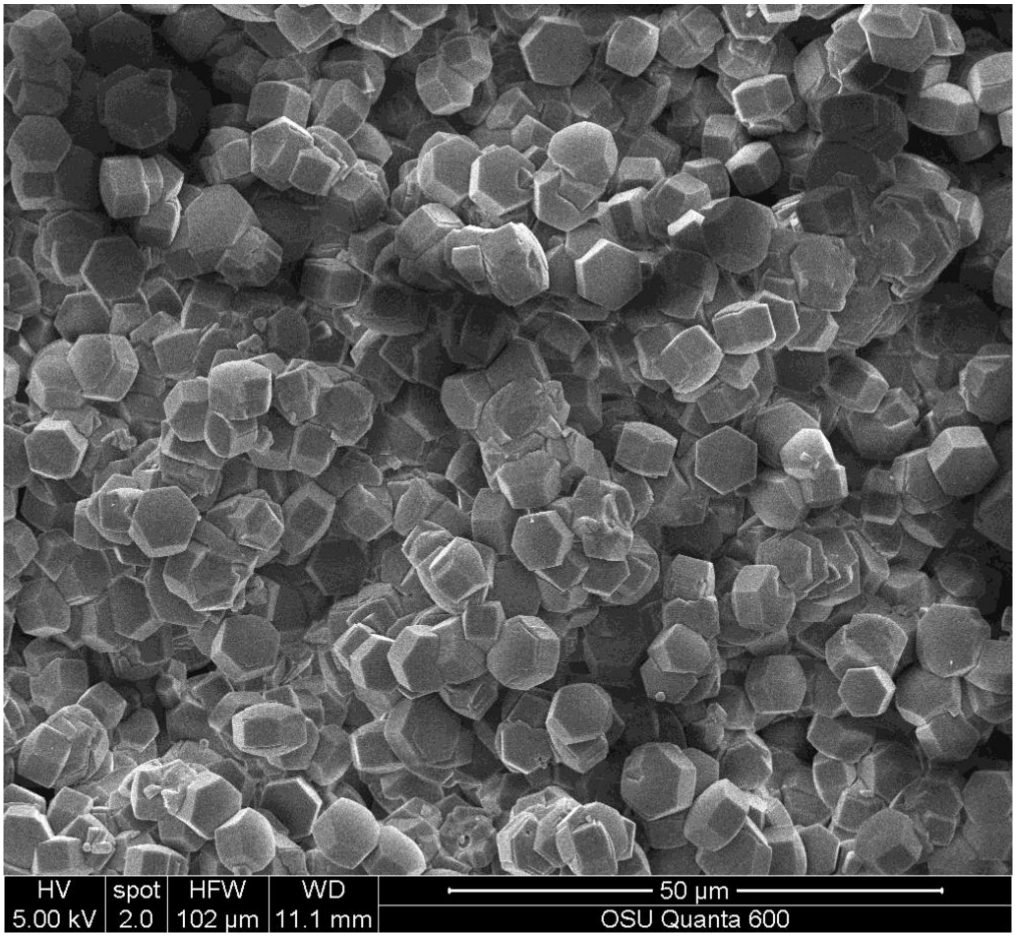

Figure S1. SEM images of crystals of as made ***m*CB-MOF-2.**

Table S1. Single Crystal X-ray Diffraction.

**Table S1.** Crystal and Structure Refinement data for ***m*CB-MOF-2**.	
Compound	***m*CB-MOF-2**
CCDC ref.	1859577
Empirical formula	C18 H16 B10 O16 Zr3
Formula weight	870.07
Crystal system	Hexagonal
Space group	*P6/mmm*
Wavelength (Å)	Synchrotron (0.72932)
Temperature	100(2)K
a (Å)	25.5012(4)
b (Å)	25.5012(4)
c (Å)	14.0513(2)
V ($Å^3$)	7913.5(3)
Z	6
$\rho_{(calc)}$ (g/cm^3)	1.095
F (000)	2532
θ range (deg)	1.763 - 28.498
Absorp. coeff. (mm^{-1})	0.626
Ind refln<br>(R_{int})	3546<br>0.0344
Goodness-of-fit on F^2	1.117
R_1 (I > 2σ(I))<br>R_1 (all data)<br>wR_2 (I > 2σ(I))<br>wR_2 (all data)	0.0503<br>0.0516<br>0.1455<br>0.1469

[a] Based on the formula without uncoordinated solvent molecules.
[b] $R_1 = \Sigma(||F_0|-|F_C||)/\Sigma|F_0|$.

[c] $wR_2 = [\Sigma w(|F_0|^2 - |F_C|^2)^2 / \Sigma w(F_0^2)]^{1/2}$.

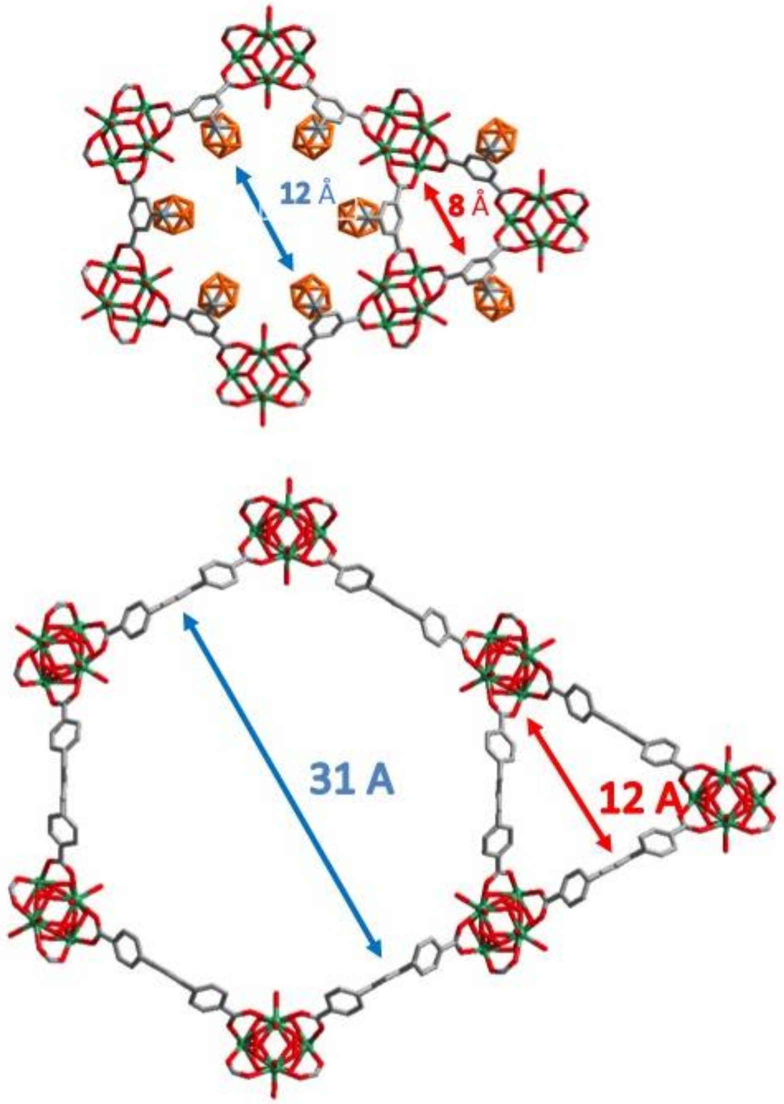

Figure S2. A comparison of the hexagonal and triangular pores for ***m*CB-MOF-2** (top) and NU-1000 (bottom). Pore diameters are indicated.

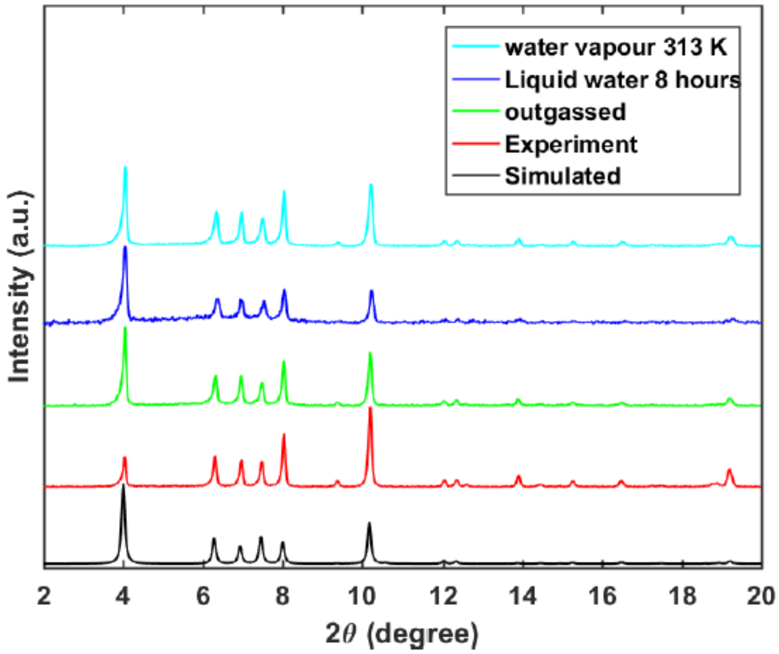

Figure S3. PXRD patterns of simulated (black), experimental (red) ***m*****CB-MOF-2**, and activated ***m*****CB-MOF-2'** (green).

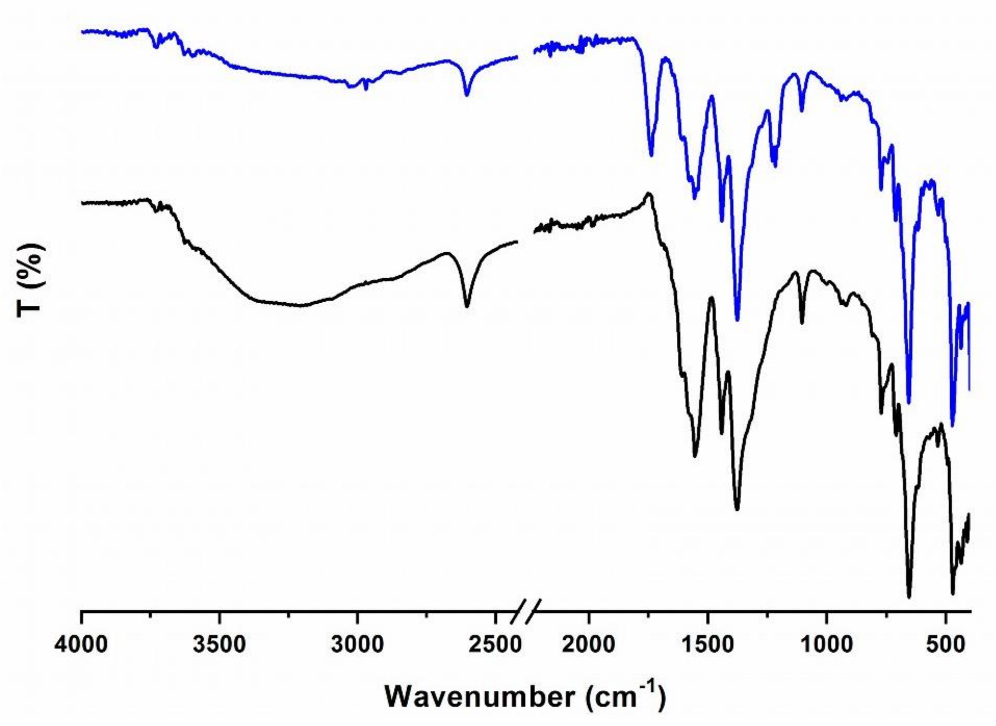

Figure S4. FT-IR spectra of as-made (black) and acetone exchanged ***m*CB-MOF-2** (blue).

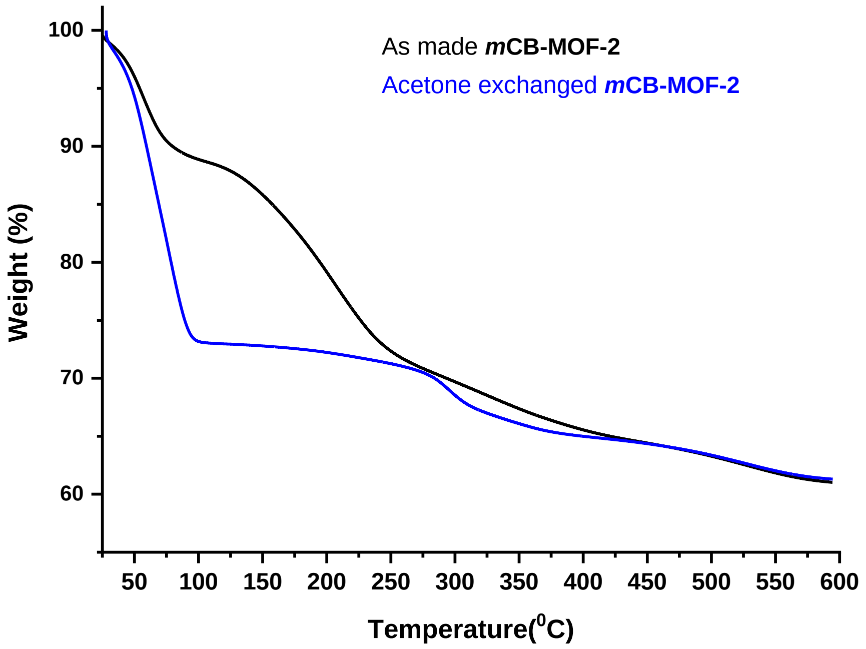

Figure S5. TGA diagrams of as-made (black) and acetone exchanged ***m*CB-MOF-2** (blue).

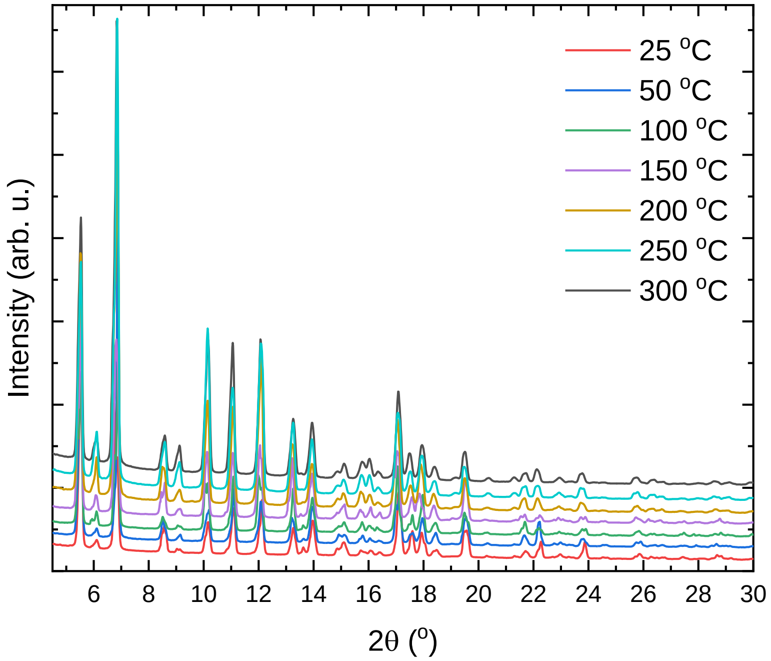

Figure S6. Variable temperature WAXS patterns of acetone exchanged ***m*CB-MOF-2** under vacuum.

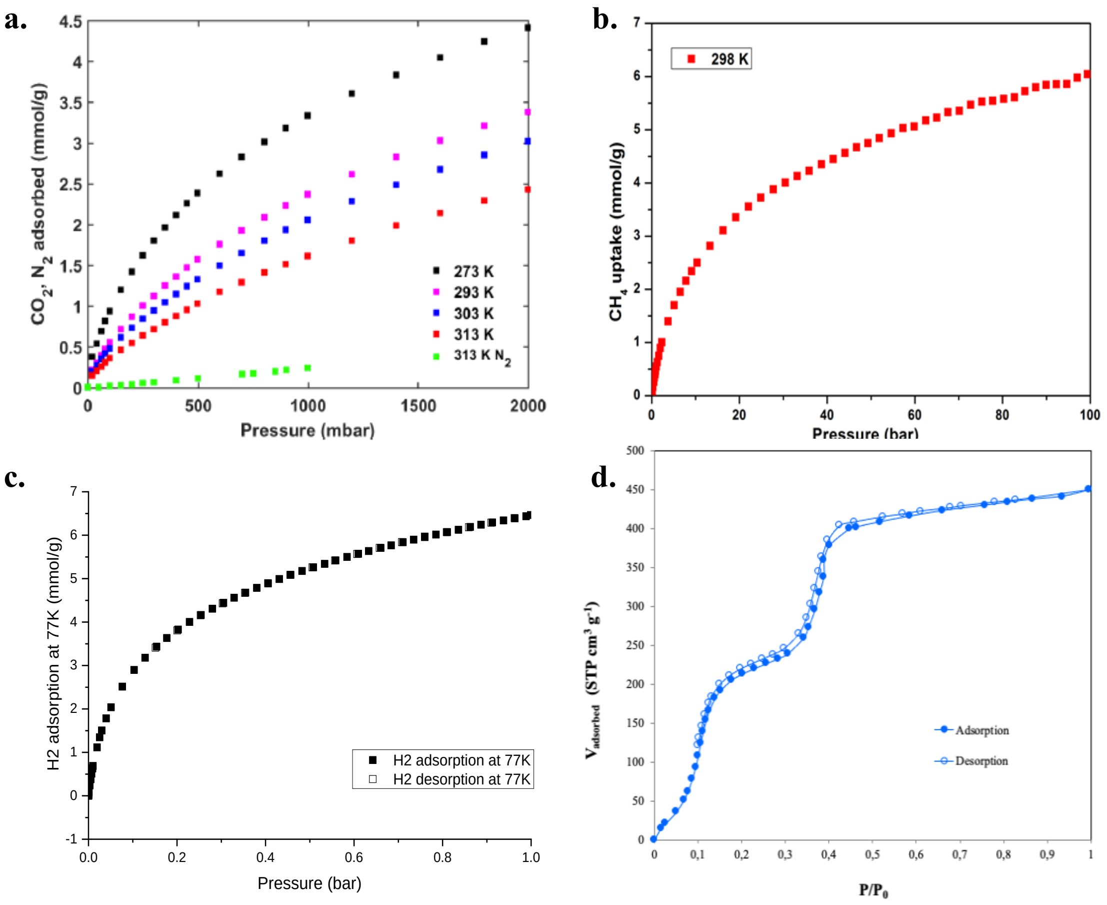

Figure S7. Adsorption isotherms for ***m*CB-MOF-2**. **a.** CO_2 adsorption isotherms at 273-313 K; **b.** CH_4 adsorption isotherms at 298K; **c.** H_2 adsorption isotherms at 77 K, and **d.** H_2O adsorption isotherms at 298K.

Table S2. Surface areas, N_2 uptakes, and total pore volumes for samples of ***m*CB-MOF-2'** treated under various conditions.

**Conditions**	**BET surface area (m^2/g)**	**N_2 uptake (STP cm^3 g^{-1})[a]**	**Pore volume (cm^3/g)[b]**
130ºC, vacuum, 12h	1095	288	0.45
2 days in water (rt)	1088	288	0.45
2 days in water (90ºC)	1024	274	0.42
2 months in water (90ºC)	1013	296	0.45
1 day, pH=1 (rt)	945	248	0.38
1 day, pH=2 (rt)	923	289	0.45
1 day, 12 M HCl	855	223	0.35
1 day, pH=11 (rt)	829	261	0.40
After GP photodegradation	995	238	0.41

[a] Measurement was taken at P/P_0 = 0.95. [b] Calculated by single point method.

When ***m*CB-MOF-2'** was immersed in acidic (pH 1) or basic (pH 11) conditions for 24 h, the N_2 uptake was mostly retained, with uptakes of 261 and 248 STP cm^3/g, respectively, as compared to the pristine sample (288 cm^3/g; Table S2). As expected, a similar trend was observed with the BET surface areas of the treated samples. ***m*CB-MOF-2'** yielded a surface area of 1095 m^2/g, and the treated MOFs showed surface areas ranging from 1088 to 829 m^2/g (Table S2).

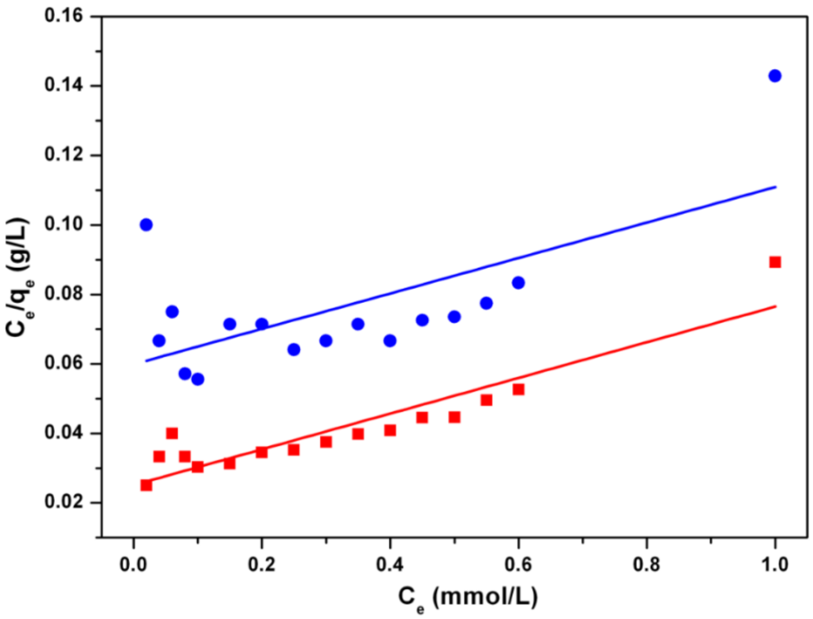

Figure S8. Langmuir Model Plots for ***m*CB-MOF-2**. The red line represents GP adsorption; the blue line represents GF adsorption.

Table S3. Langmuir and Freundlich Parameters of ***m*CB-MOF-2'** for GP and GF Adsorption.

**OPs**	**Langmuir model**			**Freundlich model**		
	**K_L (Lmmol^{-1})**	**q_m (mmolg^{-1})**	**R^2**	**K_F (mmol^{1-n} L^n g^{-1})**	**n**	**R^2**
**Glyphosate**	**2.04±0.40**	**19.46±2.21**	**0.83758**	**18.10±1.50**	**0.79±0.04**	**0.95715**
**Glufosinate**	**0.85±0.34**	**19.59±6.10**	**0.38543**	**12.43±1.25**	**0.94±0.05**	**0.95589**

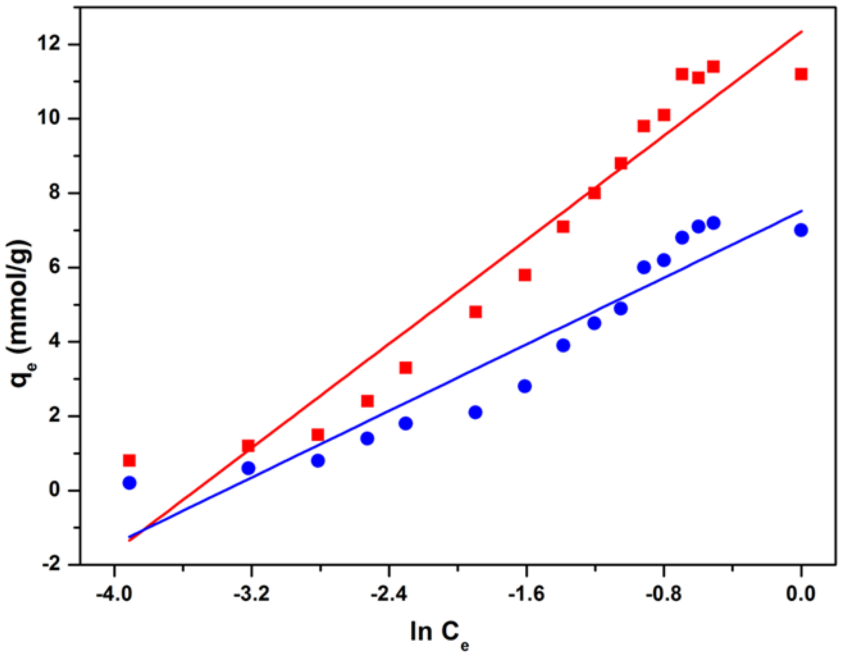

Figure S9. Temkin Model Plots for mCB-MOF-2. The red line represents GP adsorption; the blue line represents GF adsorption.

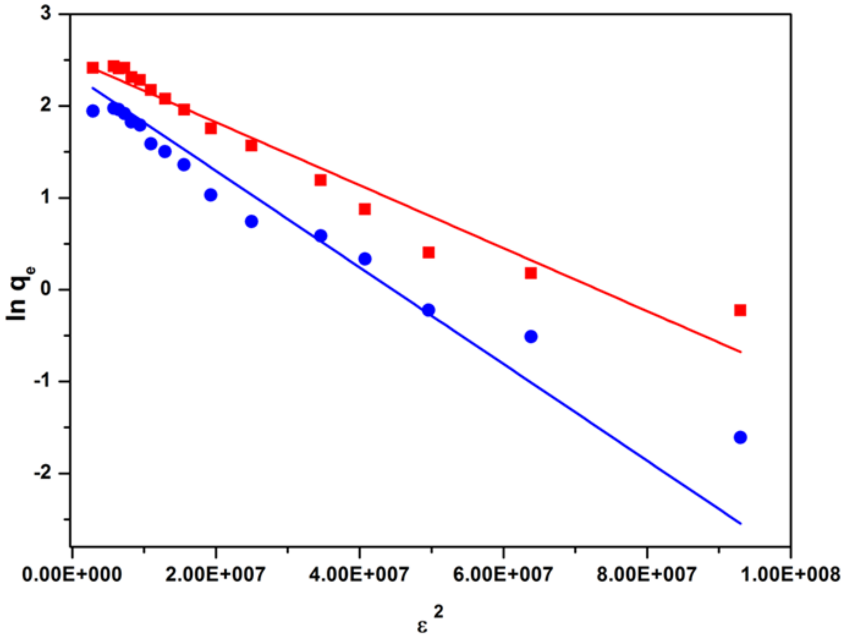

Figure S10. Dubinin-Radushkevich Model Plots for mCB-MOF-2. The red line represents GP adsorption; the blue line represents GF adsorption.

Table S4. Temkin and Dubinin-Radushkevich Parameters of mCB-MOF-2' for GP and GF Adsorption

OPs	Temkin model			Dubinin-Radushkevich model		
	A ($Lmol^{-1}$)	B ($Jmol^{-1}$)	R^2	q_m ($mmolg^{-1}$)	β (mol^2kJ^{-2})	R^2
**Glyphosate**	**34.06±5.21**	**3.49±0.24**	**0.93288**	**12.29±0.88**	**3.43E-8± 2.02E-9**	**0.95007**
**Glufosinate**	**28.69±4.97**	**2.24±0.19**	**0.90395**	**10.45±0.83**	**5.25E-8±2.15E-9**	**0.91176**

Based on the data above, the mean free energy (E) in the Dubinin-Radushkevich model can be calculated from the expression $1/\sqrt{2\beta}$ as kJ/mol and the value were 20.86 kJ/mol for GP adsorption and 16.83 kJ/mol for GF adsorption, which indicated the strong chemisorption process. However, when the correlation coefficient R^2 were compared for all fitting models, the best model to use for both GP and GF adsorption is the Freundlich model as R^2 is largest among other models.

Table S5. Comparison of the Adsorption Capacities of GP and GF onto Various Adsorbents.

OPs	Adsorbent	q_{max} (mmolg^{-1})	Reference
GP	$MnFe_2O_4$−graphene	0.23	[15]
GP	MnO_x/Al_2O_3	0.69	[16]
GP	dendro biochar	0.26	[17]
GP	chitosan/alginate membrane	4.73 X 10^{-5}	[18]
GP	polyaniline/ZSM-5	0.58	[19]
GP	montmorillonite	0.295	[20]
GP	alum sludge	0.67	[21]
GP	Ni_2AlNO_3	1.02	[22]
GP	α-FeOOH	0.23	[23]
GP	MgAl-LDH	1.09	[24]
GP	UiO-67	3.18	[25]
GP	UiO-67 (100-200 nm)	7.90	[6]
GP	Fe_3O_4@SiO_2@UiO-67	1.52	[26]
GP	UiO-67@GO	2.855	[27]
GP	NU-1000 (100-200nm)	8.97	[6]
GP	*m*CB-MOF-2’	11.4	This work
GF	UiO-67	1.98	[25]
GF	*m*CB-MOF-2’	7.2	This work

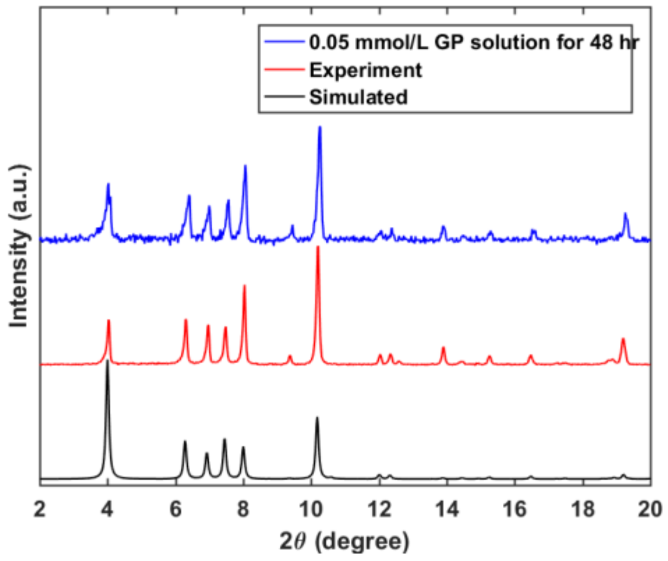

Figure S11. PXRD patterns of ***m*CB-MOF-2** after GP adsorption.

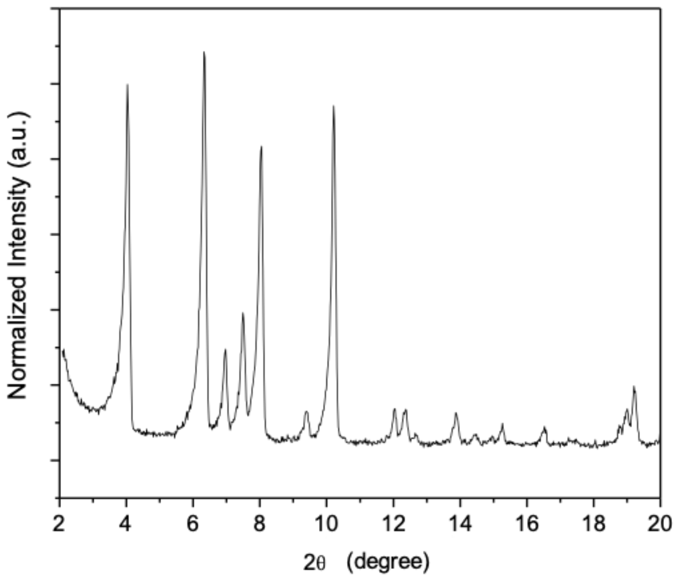

Figure S12. PXRD patterns of ***m*CB-MOF-2** after GF adsorption.

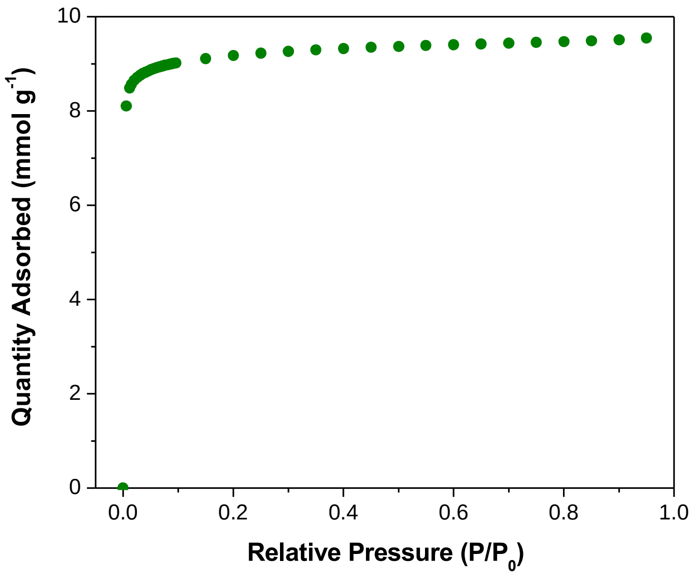

Figure S13. N_2 adsorption collected at 77K for ***m*CB-MOF-2** after GP uptake. ***m*CB-MOF-2** was regenerated after washing with acidified water for three times to remove any GP from the pores, and activated at 60 ^oC for 4 h. BET surface area was calculated to be 997 m^2/g.

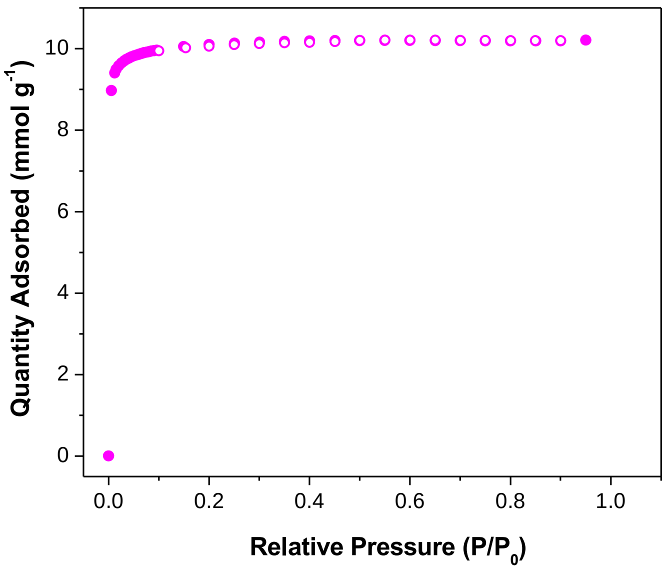

Figure S14. N_2 adsorption collected at 77K for ***m*CB-MOF-2** after GF uptake. ***m*CB-MOF-2** was regenerated after washing with acidified water for three times to remove any GF from the pores, and activated at 60 ^oC for 4 h. BET surface area was calculated to be 1071 m^2/g.

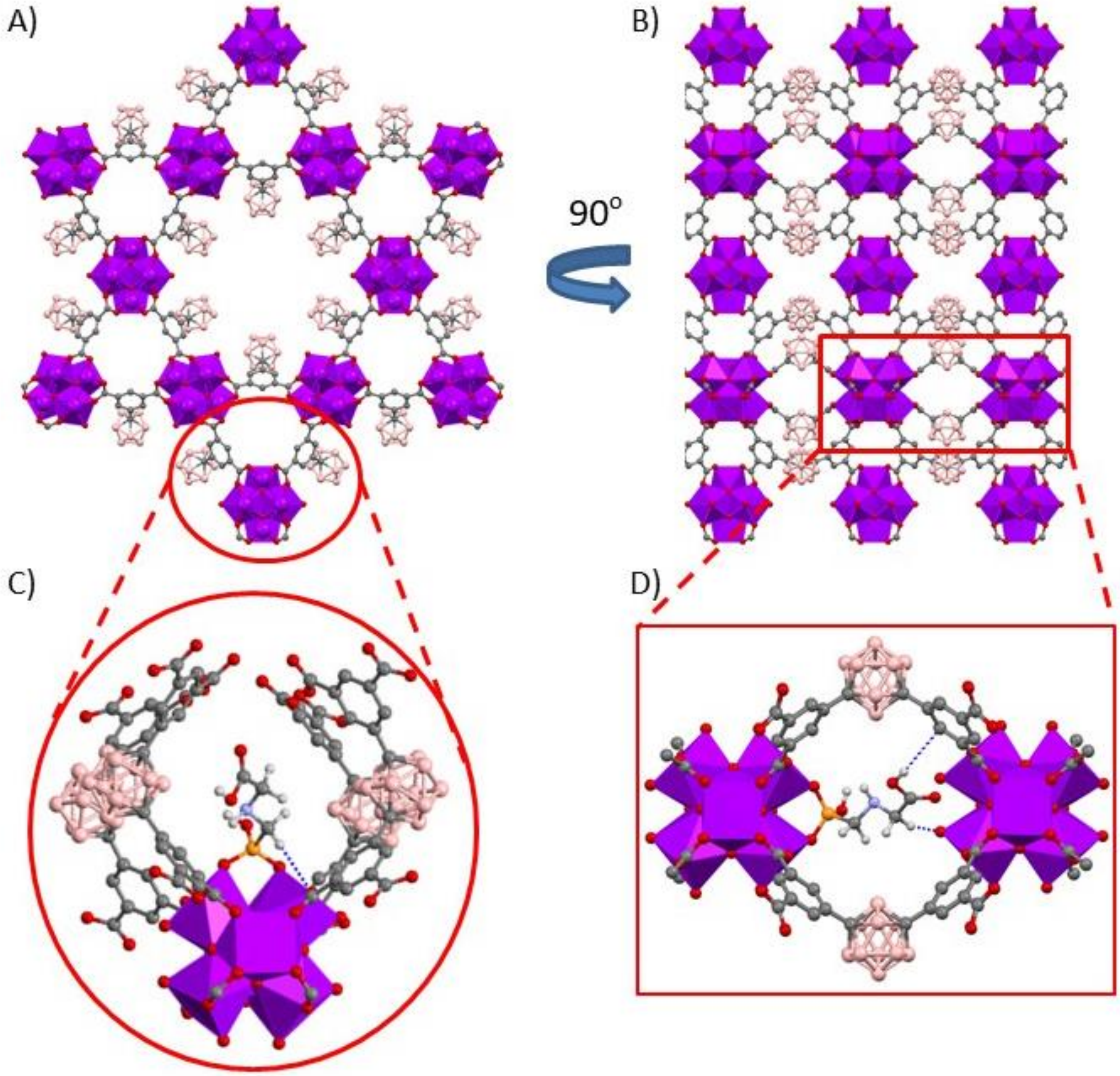

Figure S15. 3D structure of ***m*CB-MOF-2** showing (A) the hexagonal and triangular pores along the *c*-axis and (B) c-pores along the *b*-axis (approx. window of 6 x 3 Å). Lowest energy conformations of glyphosate binding (C) in the triangular pores of ***m*CB-MOF-2** showing noncovalent interaction with the surrounding framework environment as a blue dotted line (C–H···O: H···O 2.855 Å, CHO 134°) and (D) in the c-pores of ***m*CB-MOF-2** showing noncovalent interaction with the surrounding framework environment as blue dotted lines (C–H···O: H···O 2.196 Å, CHO 162°; O–H···C: H···C 2.884 Å, OHC 126°).

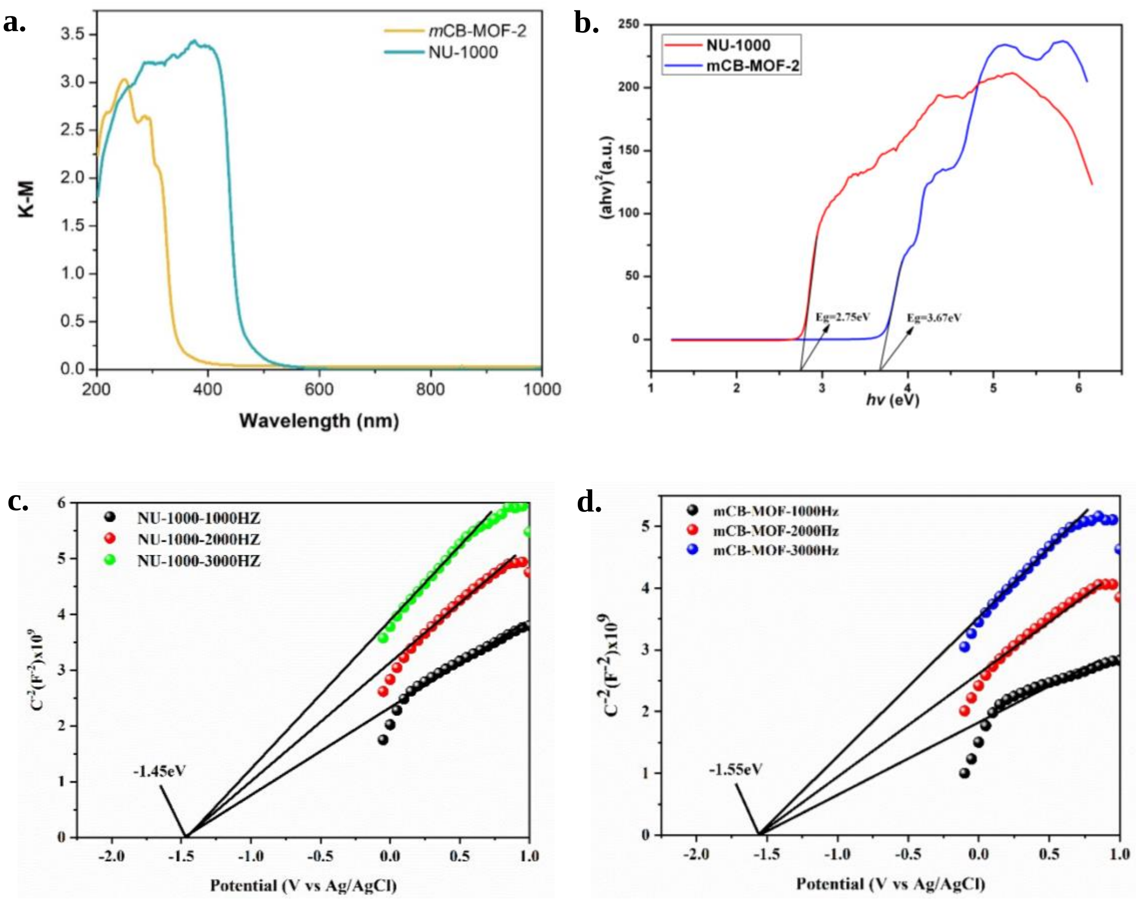

Figure S16. a. UV-vis diffuse reflectance spectra for ***m*CB-MOF-2** and NU-1000, **b.** Tauc plots, **c. and d.** Mott–Schottky plots in 0.5 M Na_2SO_4 aqueous solution at 1000, 2000, and 3000 Hz for NU-1000 and ***m*CB-MOF-2**.

Table S6. Chemical shift values (ppm), splitting patterns, and labels for ^1H and ^{31}P NMR.

Compound	^1H (ppm)*	^{31}P (ppm)	Label
Glyphosate	3.2605, 3.2351 (d)<br>3.9285 (s)	8.7120	A<br>B
Sarcosine	2.7245 (s)<br>3.5981 (s)	N/A	C<br>D
AMPA	3.0944, 3.0689 (d)	10.8183	E
Acetic acid	2.0794 (s)	N/A	F
Formic acid	8.23 (s)	N/A	G
Orthophosphate	N/A	0.0038	None

* Letter in parenthesis denotes splitting pattern, singlet (s) or doublet (d).

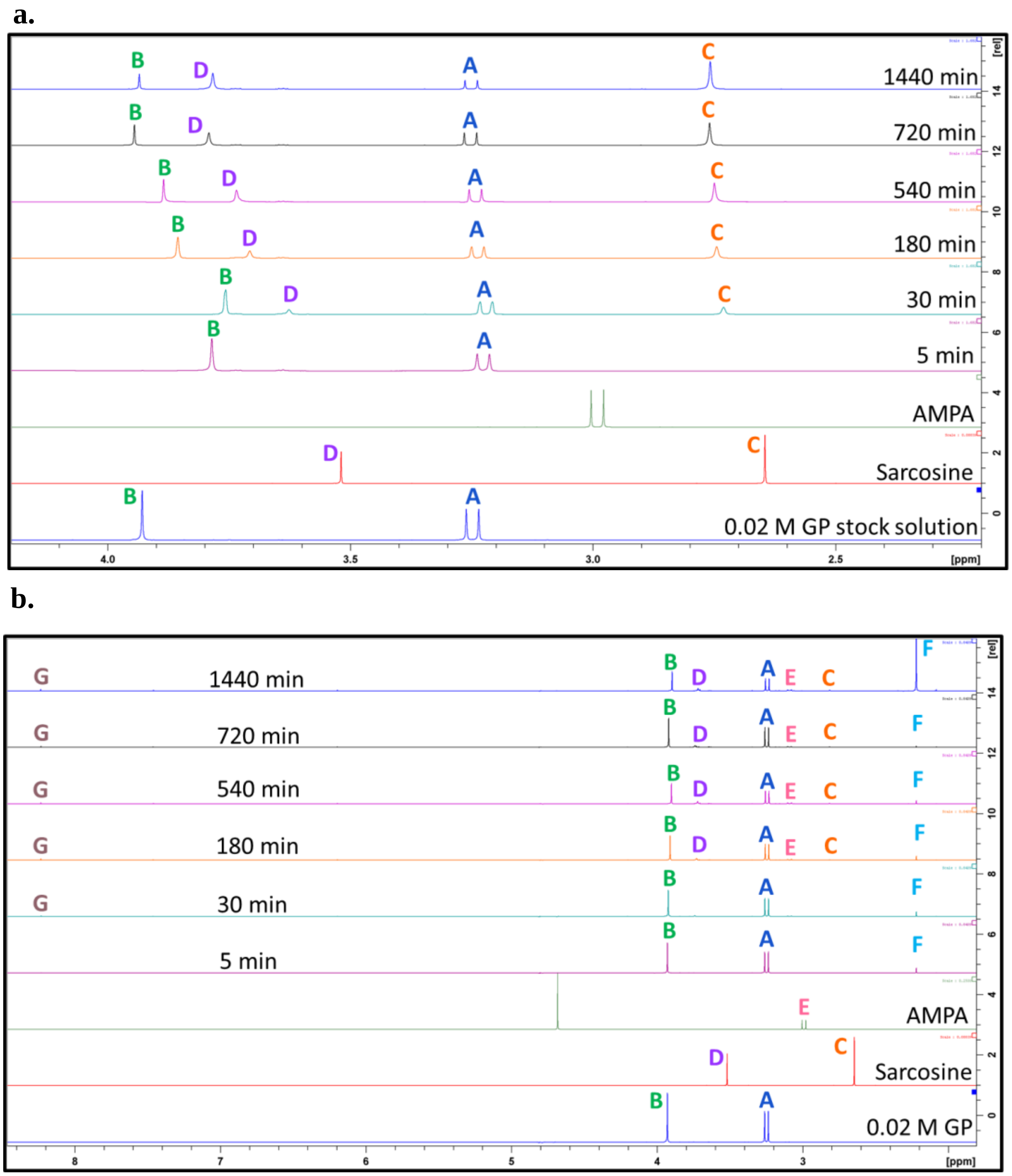

Figure S17. ^{1}H NMR spectra for each GP photodegradation solution using ***m*****CB-MOF-2** (**a.**) or TiO_2 (**b.**). Only sarcosine [δ 2.57 (s) and δ 3.57 (s)] and GP [δ 3.06, 3.07 (d) and δ 3.71 (s)] are seen in (**a.**) for ***m*****CB-MOF-2**, while all GP [δ 3.15 and 3.17 (d) and δ 3.82 (s)], sarcosine [δ 2.73 (s) and δ 3.64 (s)] and AMPA [δ 2.99 and 3.02 (d)] are seen for TiO_2 (**b.**). Shifts in the peak positions were attributed to the changing pH throughout the irradiation due to the production of orthophosphate, PO_4^{3-}). Peaks F and G correspond to the production of acetic and formic acid respectively.

a.

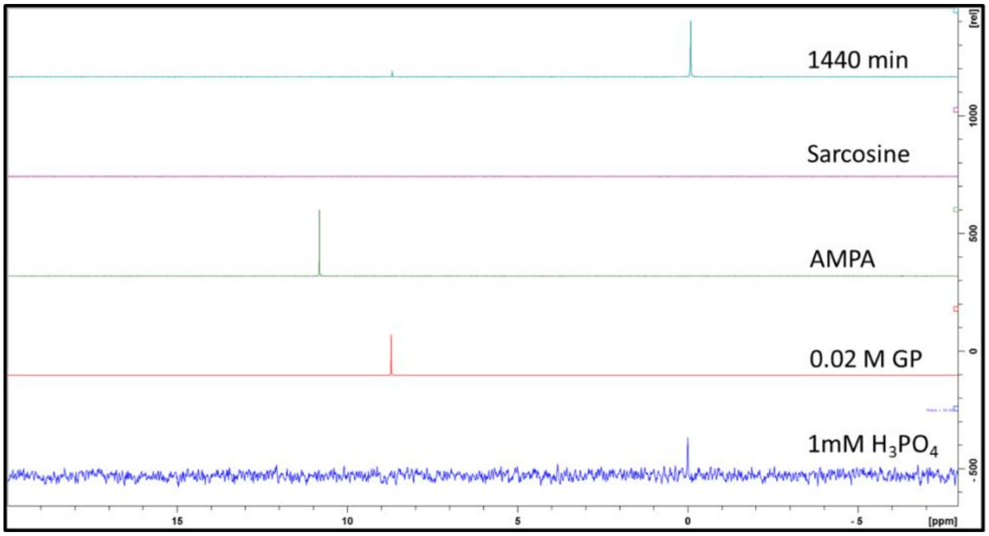

b.

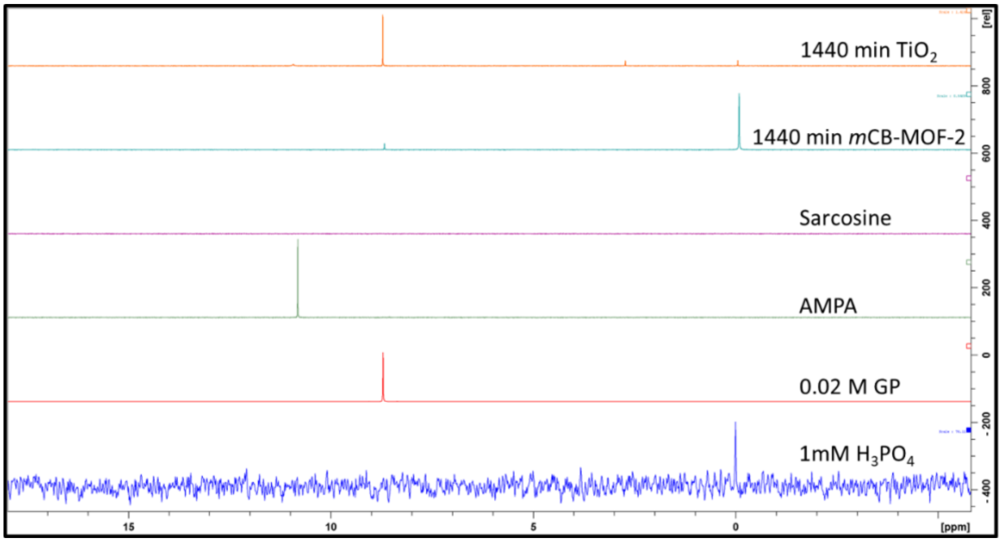

Figure S18. ^{31}P-NMR of GP photodegradation solution using **a.** ***m*CB-MOF-2** or **b.** TiO_2. In **a.**, there is no evidence of AMPA in the ^{31}P spectrum for ***m*CB-MOF-2**, while in **b.**, the ^{31}P peak corresponding to AMPA can be seen at ~10.82 ppm. The large peak at approximately 0.00 ppm for corresponds to orthophosphate, which helps support the C-P Lyase type degradation by ***m*CB-MOF-2** and our hypothesis that the shifting peak positions are due to pH changes throughout the reaction. Additionally, a large peak for orthophosphate is observed for ***m*CB-MOF-2** (**a.**), while the same peak for TiO_2 (**b.**) is much smaller, demonstrating again that ***m*CB-MOF-2** is selective in forming only sarcosine and orthophosphate.

Upon thoroughly investigating the ^{1}H NMR data for the photodegradation of GP via ***m*CB-MOF-2**, we noticed that longer UV-Vis irradiation times resulted in a downfield shift of the peaks corresponding to the α-protons for both GP and sarcosine. As sodium 3-(trimethylsilyl)-1-propane sulfonate (DSS) was used as an internal standard (Figure S13) and to calibrate the axes, the shifting peak positions are likely due to other changes, such as pH (i.e., the production of orthophosphate). Changes in pH throughout the reaction would result in changes in the chemical shift environment (electronic environment) of the α-protons. Previous work has shown that these changes can either be through-bond or through-space.[28] While the downfield shifting behavior remained consistent among all ***m*CB-MOF-2** catalyzed reactions, TiO_2-catalyzed reactions showed only minor changes in peak positions (Figure S13b). The differences in the degree of shifting for each photocatalyst might be attributed to the: (i) concentration of the products formed after irradiation; (ii) presence of products that could change the pH; and (iii) various protonated forms of GP and sarcosine in acidic solutions. To better understand the effect of the pH on the NMR peaks, ^{1}H NMR experiments were conducted with GP and sarcosine using varying amounts of H_3PO_4 (1.0 mM) or potassium hydroxide (KOH 1.0 M) to simulate acidic or basic conditions, respectively. The resulting shifts for the simulated acidic environment with H_3PO_4 were downfield, while insignificant shifts were observed for the simulated basic environment with KOH.

Sarcosine exists primarily in its zwitterionic form up to pH 7.3.[29] At pH 7.3 and below, the secondary amine of sarcosine is doubly protonated, and the first pKa of sarcosine is 2.2 for the fully protonated carboxylic acid.[30] Qualitative investigation of the pH of the reaction solution after 12 h irradiation using ***m*CB-MOF-2** showed a slightly acidic pH ~ 3, while TiO_2 had an acidic pH ~2. The pH of the 0.02 M GP solution is already acidic, pH ~2. Considering the zwitterionic nature of sarcosine, the shielding and de-shielding effects in ^{1}H NMR, and the changing pH conditions throughout the reaction, we propose that the downfield shift in the sarcosine peak at ~3.3 ppm in the ^{1}H NMR for ***m*CB-MOF-2** is because the carboxylate end of GP (at pH ~3) de-shields the α-protons.

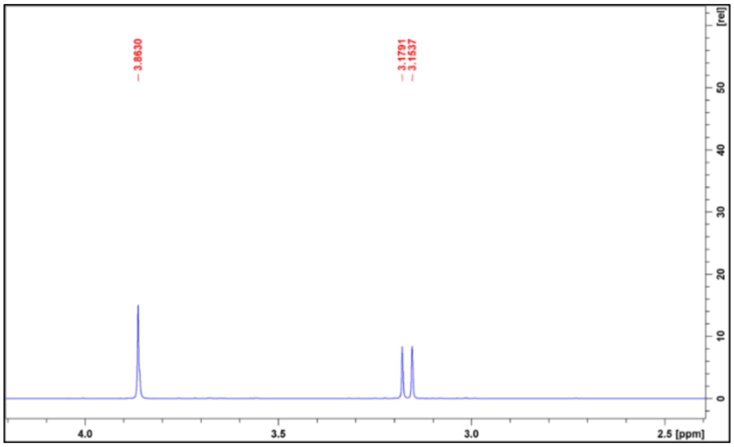

Figure S19. ^{1}H NMR of the 0.02 M glyphosate solution after a 24 h irradiation (without any catalyst) shows no degradation products, indicating it does not photodegrade without a catalyst under our experimental conditions.

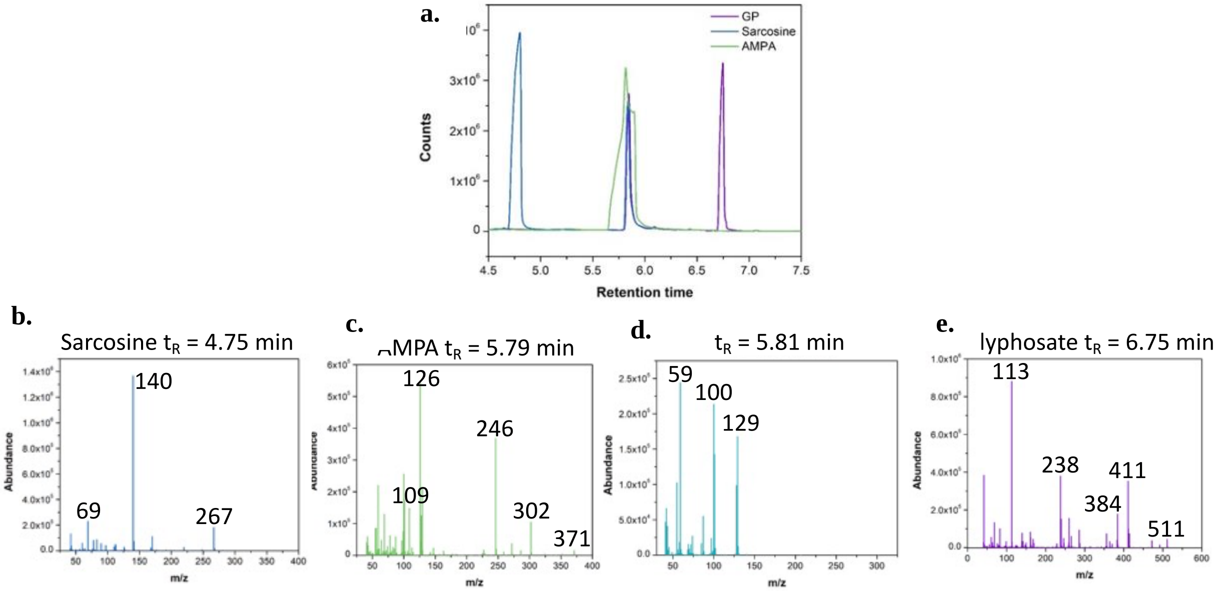

Figure S20. GCMS results for derivatized standards, **a.** Total ion chromatogram (TIC), **b.** mass spectrum for derivatized sarcosine (major ion of derivatized sarcosine *m/z* 267 and intense ion at *m/z* 140 from loss of CH_3NCOCF_3); **c.** mass spectrum for derivatized AMPA (major ion of derivatized AMPA *m/z* 371 and another at 302 from loss of CF_3); **d.** unknown compound, likely a byproduct of the derivatization process, as it is present in both the derivatized standards and samples. It has an elution time of 5.81 min (co-elutes with AMPA) and a 91% match in the mass spec library for dimethyl glutarate ($C_7H_{12}O_4$); **e.** mass spectrum for derivatized glyphosate (major ion of derivatized GP *m/z* 511 and another peak at 411 from loss of TFE).

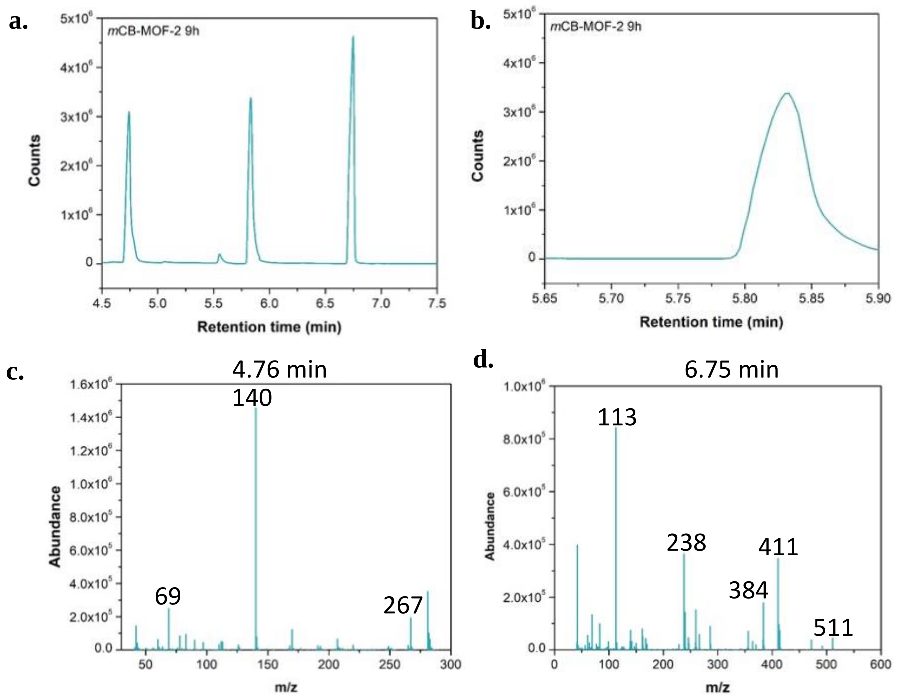

Figure S21. GCMS data for ***m*CB-MOF-2**, 9 h GP irradiation. **a.** TIC showing the presence of sarcosine (t_R = 4.72 min), an unknown fluorinated amide (t_R = 5.55 min), an unknown byproduct, likely dimethyl glutarate (t_R = 5.81 min), and glyphosate (t_R = 6.78 min); **b.** Zoomed in TIC around the expected retention time of AMPA, showing that no peak for AMPA was observed (t_R = 5.79 min); **c.** and **d.** showing the mass spectra for the sarcosine and glyphosate peaks with expected major ions present.

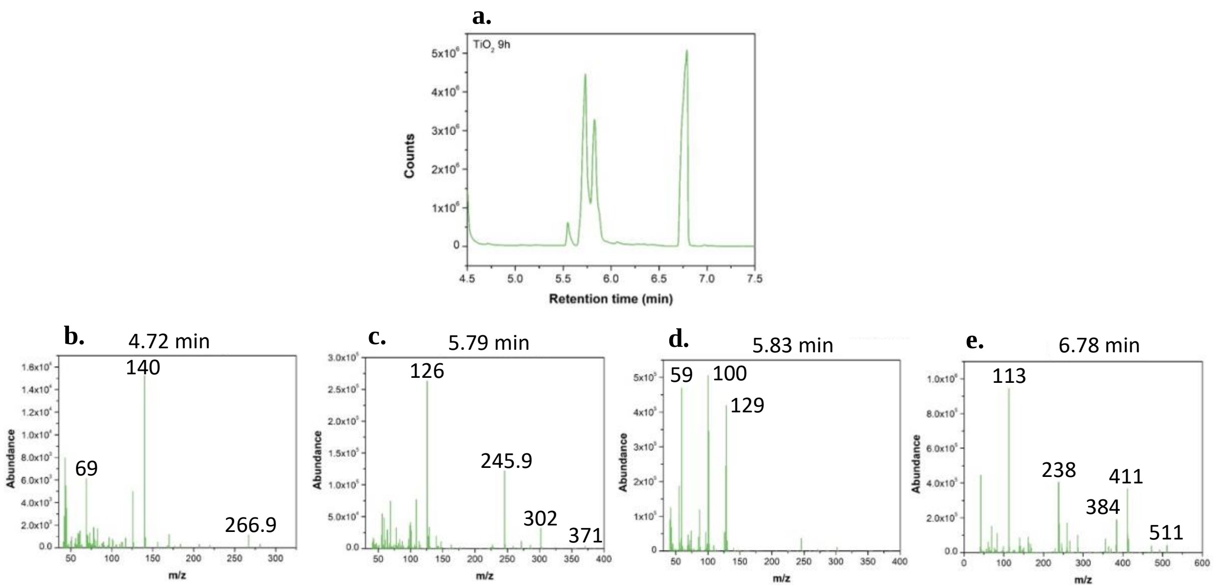

Figure S22. GCMS for TiO_2, 9 h GP irradiation. **a.** TIC indicates a small peak for sarcosine (t_R = 4.72 min), the unknown fluorinated amide (t_R = 5.55 min), AMPA (t_R = 5.79 min), unknown byproduct, likely dimethyl glutarate (t_R = 5.83 min) and glyphosate (t_R = 6.78 min). **b.**, **c.**, **d.**, and **e.** are the mass spectra representing each retention time with the expected major ion peaks for sarcosine, AMPA, and glyphosate.

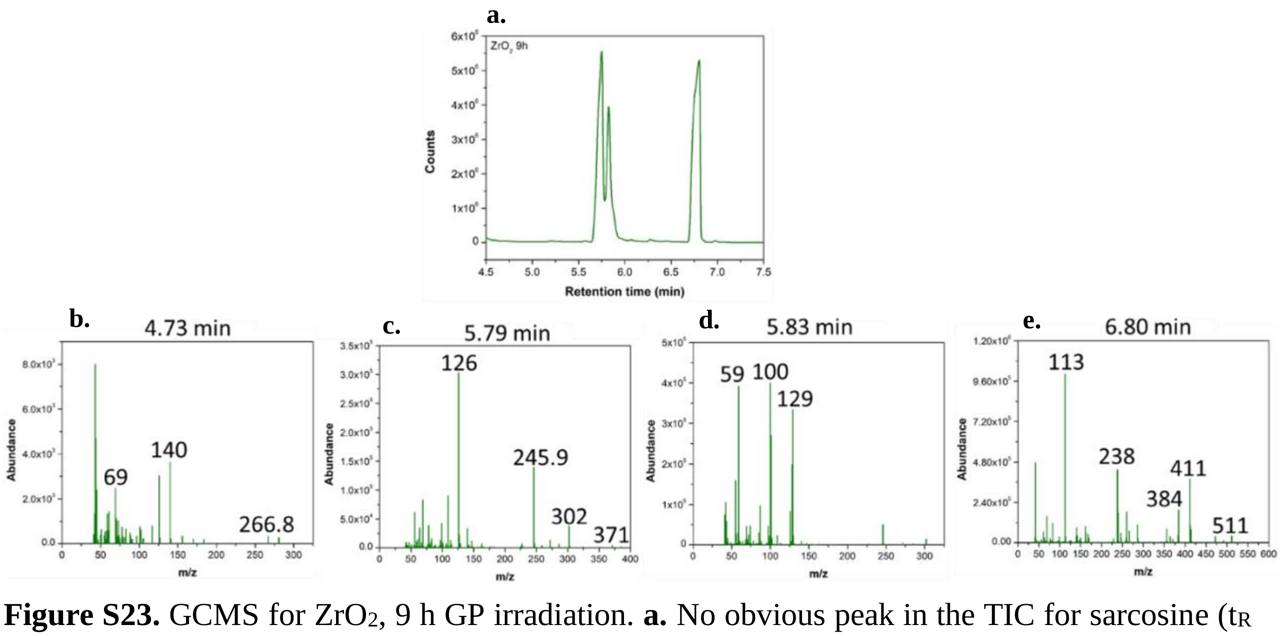

Figure S23. GCMS for ZrO_2, 9 h GP irradiation. **a.** No obvious peak in the TIC for sarcosine (t_R = 4.73 min), but the mass spectrum (**b.**) for 4.73 min indicates some *m/z* fragments for sarcosine, AMPA (t_R = 5.79 min), unknown byproduct, likely dimethyl glutarate (t_R = 5.83 min) and

glyphosate (t_R = 6.80 min). **b.**, **c.**, **d.**, and **e.** are the mass spectra representing each retention time with the expected major ion peaks for sarcosine, AMPA, and glyphosate.

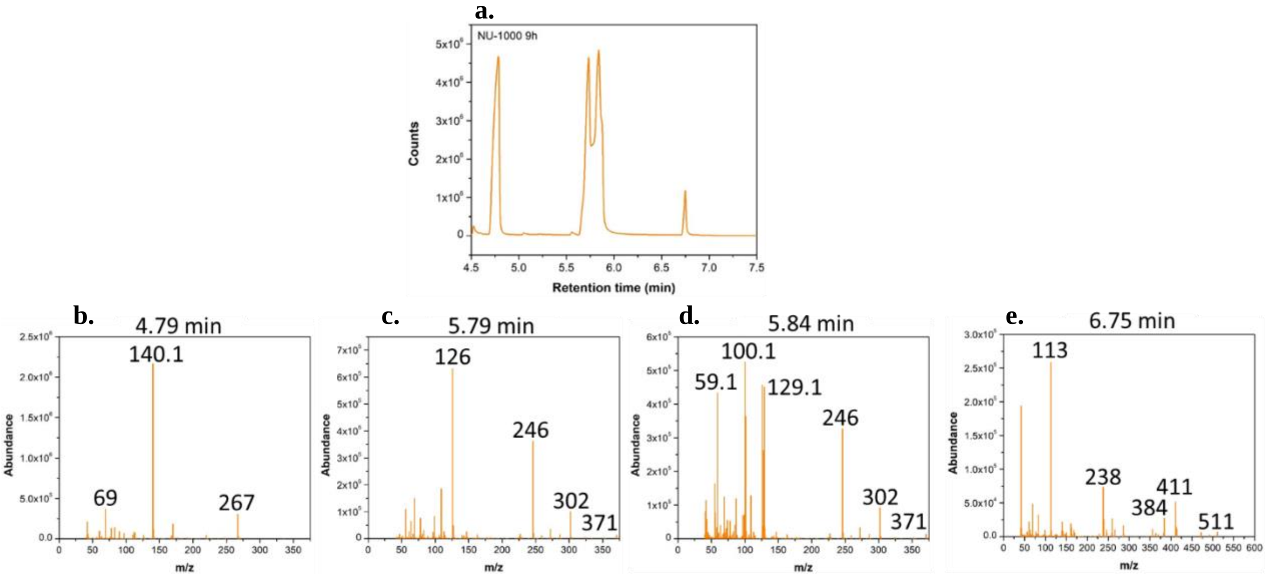

Figure S24. GCMS for NU-100, 9 h GP irradiation. **a.** TIC showing the presence of sarcosine (t_R = 4.79 min), AMPA (t_R = 5.79 min) co-eluting with the unknown derivatization byproduct, likely dimethyl glutarate (t_R = 5.84 min), and glyphosate (t_R = 6.75 min). **b.**, **c.**, **d.**, and **e.** are the corresponding mass spectra for each peak with the expected major ions for each product.

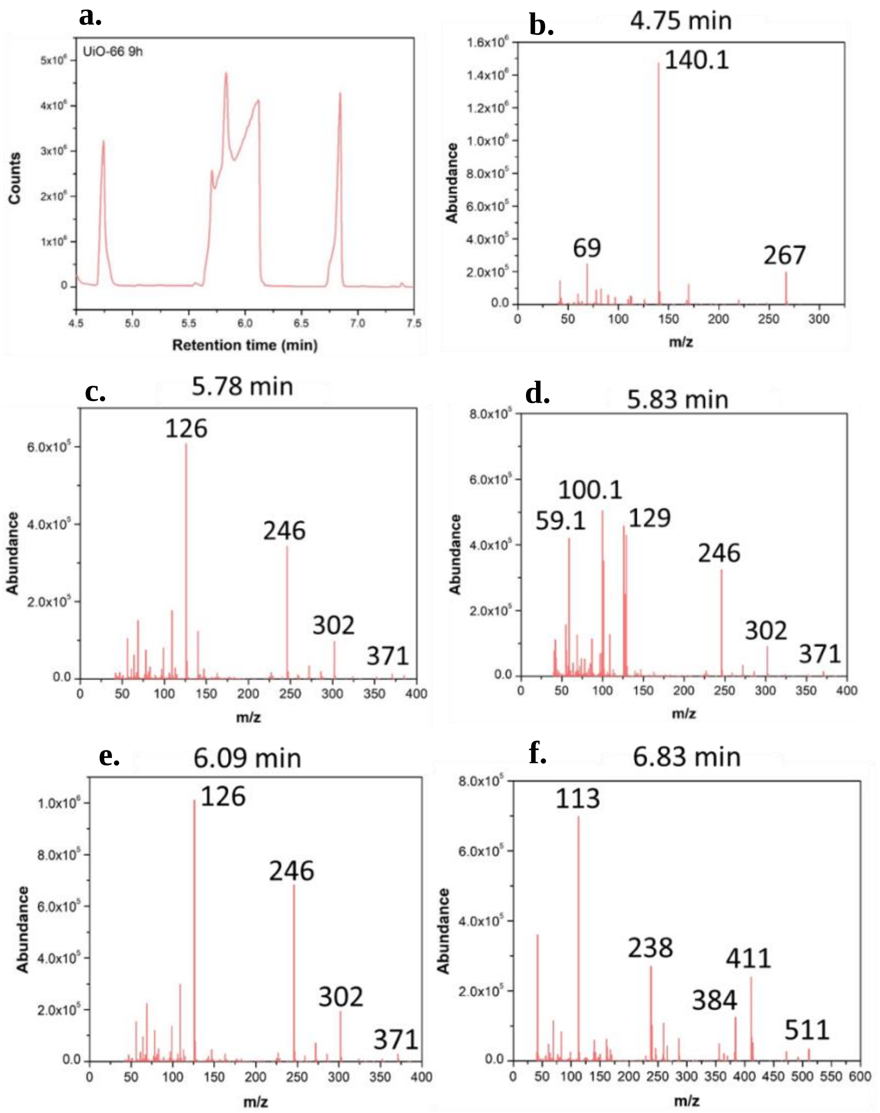

Figure S25. GCMS for UiO-66, 9 h GP irradiation. **a.** TIC showing the presence of sarcosine (t_R = 4.75 min), AMPA (t_R = 5.78 min) co-eluting with the unknown derivatization byproduct, likely dimethyl glutarate (t_R = 5.83 min), more AMPA at t_R = 6.09 min, and glyphosate (t_R = 6.75 min). **b.**, **c.**, **d.**, **e.**, and **f.** are the corresponding mass spectra for each peak with the expected major ions for each product.

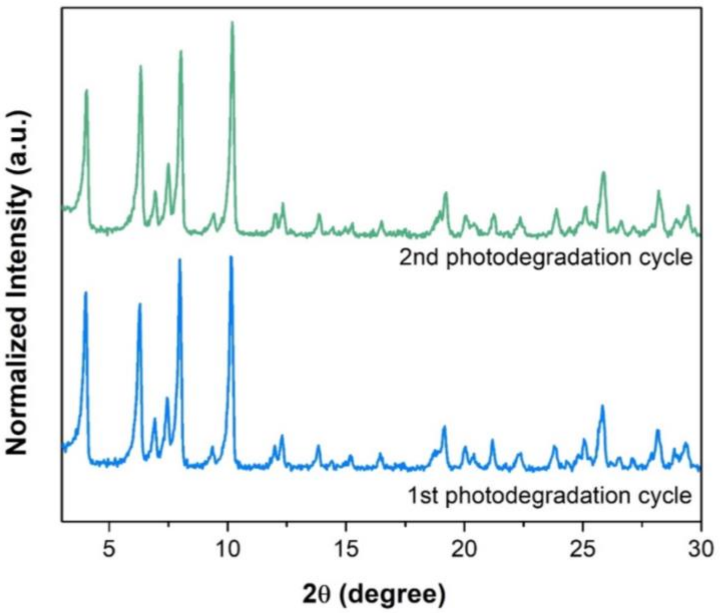

Figure S26. PXRD patterns of ***m*CB-MOF-2** after UV-Vis irradiation shows that crystallinity is maintained after UV-Vis exposure.

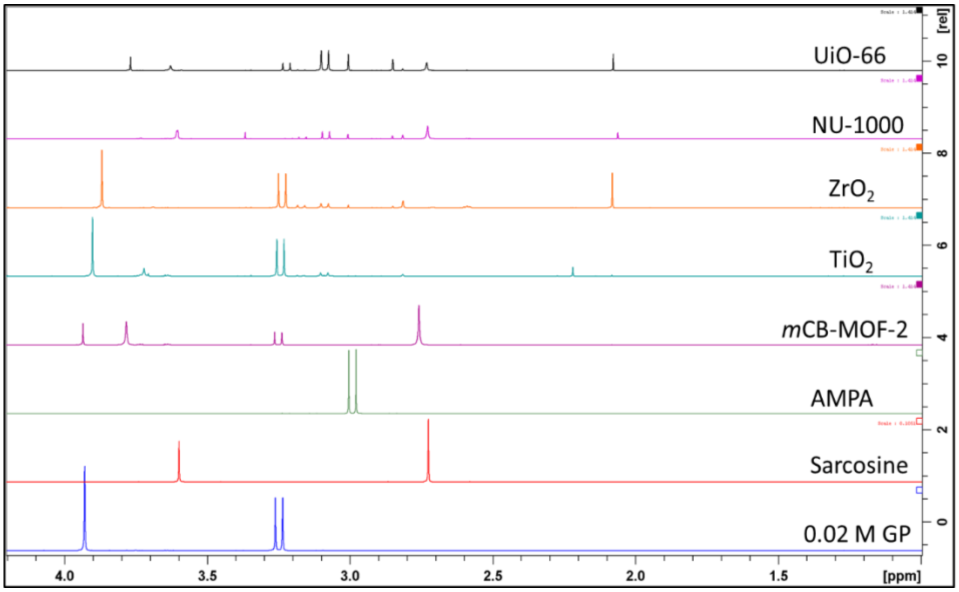

Figure S27. ^{1}H-NMR data of the GP photodegradation solution after 9 h for each photocatalyst tested. Only sarcosine peaks are seen for ***m*CB-MOF-2**, while AMPA and acetic acid peaks can be seen for the other photocatalysts.

Table S7. Summary of retention times and major ions in mass spectra of derivatized standards for GCMS.

Compound	Retention time (min)	m/z of major ions
Sarcosine	4.75	267, 140, 69
Unknown fluorinated amide	5.56	152, 139, 114, 82, 67
AMPA	5.79	302, 246, 126, 109
Dimethyl glutarate	5.82	100, 59, 42
Glyphosate	6.80	511, 411, 384, 238, 113

Table S8. Summary of ICP-OES results for AMPA uptake.

Sample	Time (min)	Concentration [P] (mg/L)	C_0-C_i (mmol/L)
200 ppm AMPA stock	N/A	198.9	N/A
*m*CB-MOF-2	5	32.2	1.50
	10	25.3	1.56
	30	24.5	1.57
	60	26.2	1.55
TiO_2	5	180.9	0.16
	10	176.9	0.20
	30	177.8	0.19
	60	169.8	0.26
NU-1000	5	12.9	1.67
	10	15.2	1.65
	30	14.7	1.66
	60	13.5	1.67

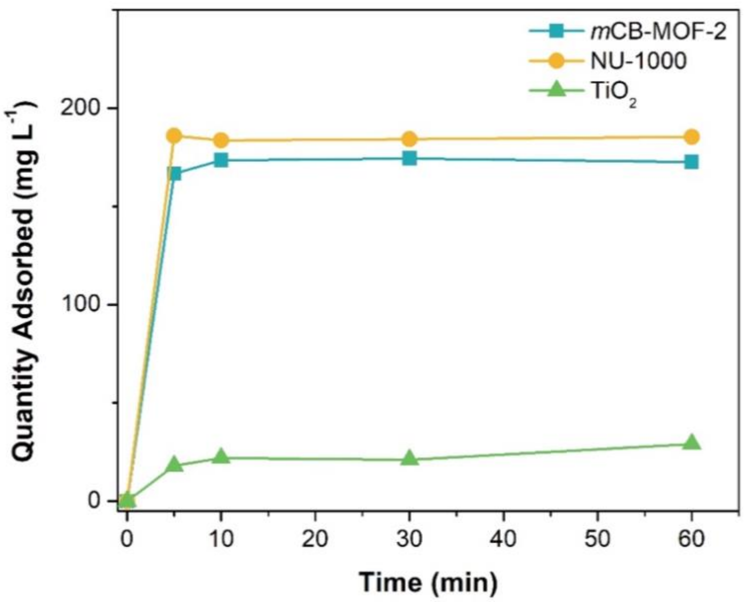

Figure S28. Adsorption of AMPA (200 ppm) on ***m*CB-MOF-2**, NU-1000, and TiO_2 after 1 h. NU-1000 shows the steepest uptake and largest adsorption (186.5 mg/L), followed by ***m*CB-MOF-2** (173.8 mg/L) and TiO_2 (30.2 mg/L).

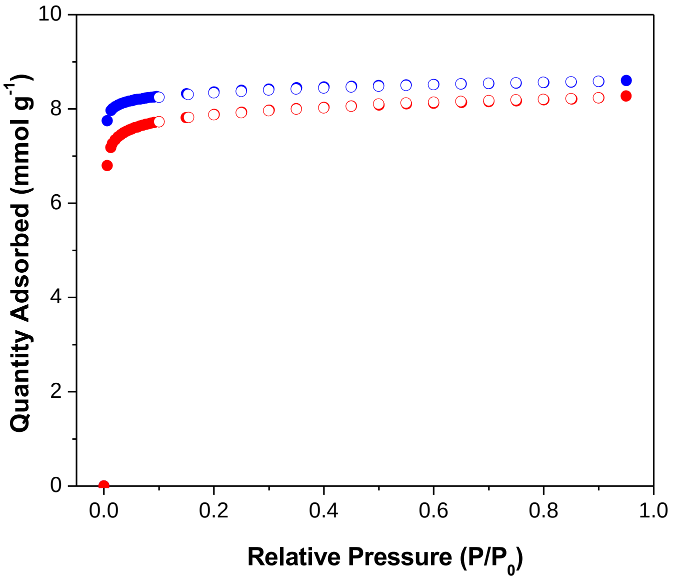

Figure S29. N_2 isotherms collected at 77K for ***m*CB-MOF-2** after AMPA uptake (red) and photodegradation (blue). BET surface area after AMPA capture decreased from 1095 m^2/g to 807 m^2/g and 916 m^2/g after AMPA photodegradation (1 h).

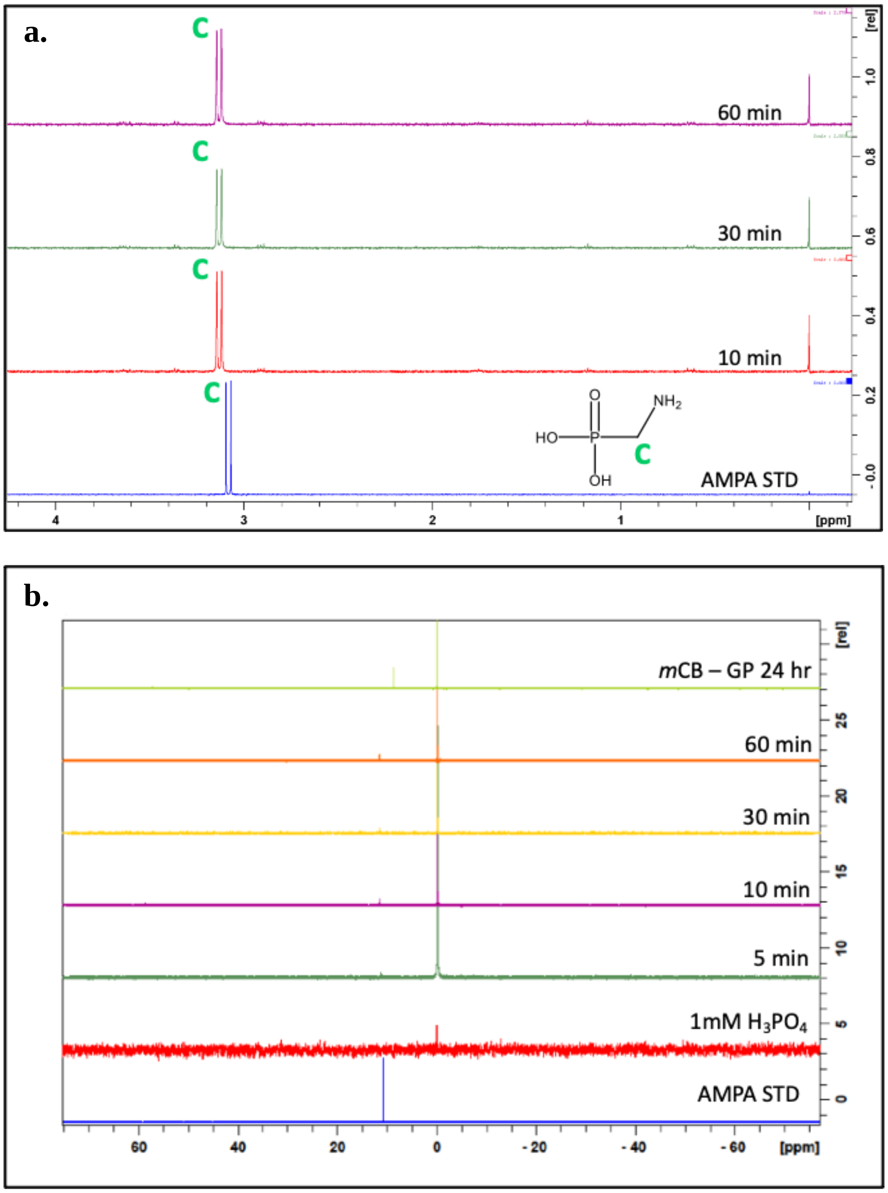

Figure S30. a. ^{1}H and **b.** ^{31}P-NMR for ***m*CB-MOF-2** after AMPA photodegradation. While we did not observe a large decrease in the intensity of the AMPA peaks in the ^{1}H-NMR spectra, the presence of orthophosphate in the ^{31}P spectra show that some AMPA is being degraded.

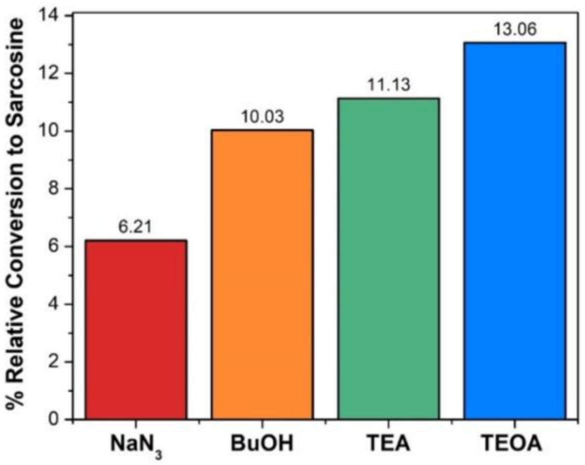

Figure S31. Photocatalytic degradation of GP with ***m*CB-MOF-2** after 9 h in the presence of various ROS scavengers. Sodium azide (NaN_3), triethylamine (TEA), triethanolamine (TEOA), and *tert*-butanol (BuOH) are commonly employed as radical scavengers for singlet oxygen ($^1O_2$), superoxide ($\cdot O_2^-$), hole (h^+) and hydroxide ($\cdot OH$), respectively. Sodium azide was used twice with comparable results (the average of two trials is shown). The low conversion of GP when NaN_3 is used suggests that $^1O_2$ is sufficiently scavenged, causing a less efficient conversion of GP to sarcosine.

Electron Spin Resonance (ESR) spectroscopy

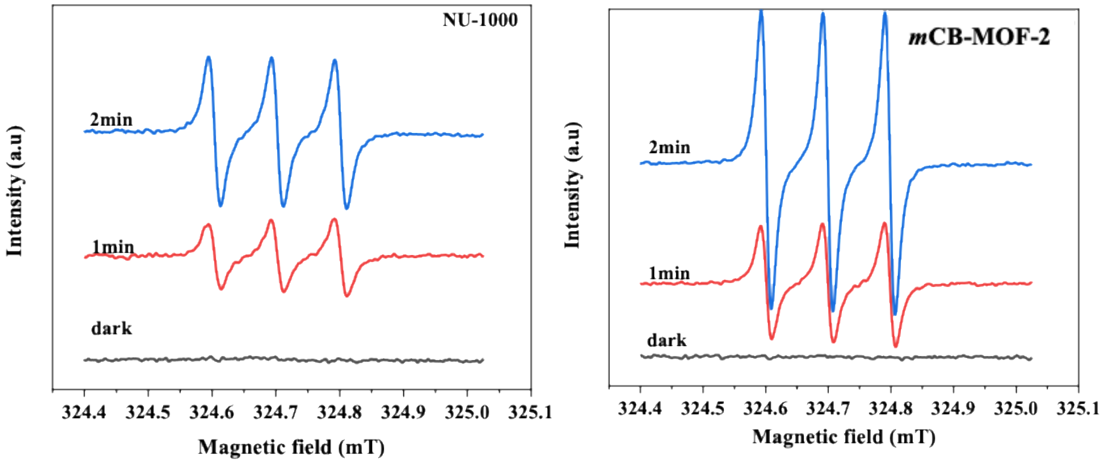

Figure S32. ESR spectra of free radicals trapped by TEMPO in the reaction with NU-1000 (left) and ***m*CB-MOF-2** (right) in the dark and under UV light irradiation in water for 1 and 2 min.

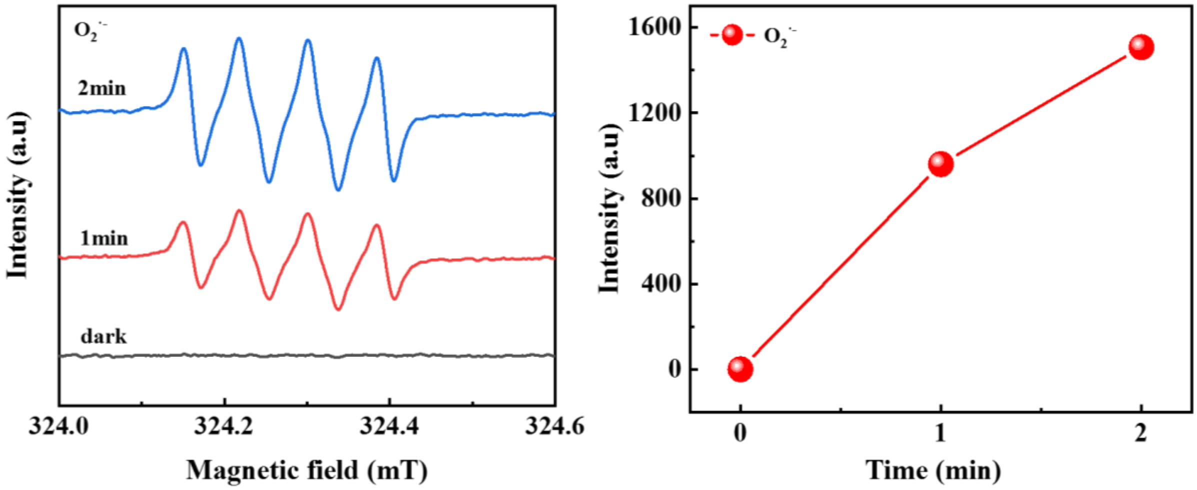

Figure S33. ESR spectra of free radicals trapped by DMPO (left) in the reaction with ***m*CB-MOF-2** in the dark and under UV light irradiation in methanol for 1 and 2 min and $O_2^{\bullet-}$ generation rate (right).

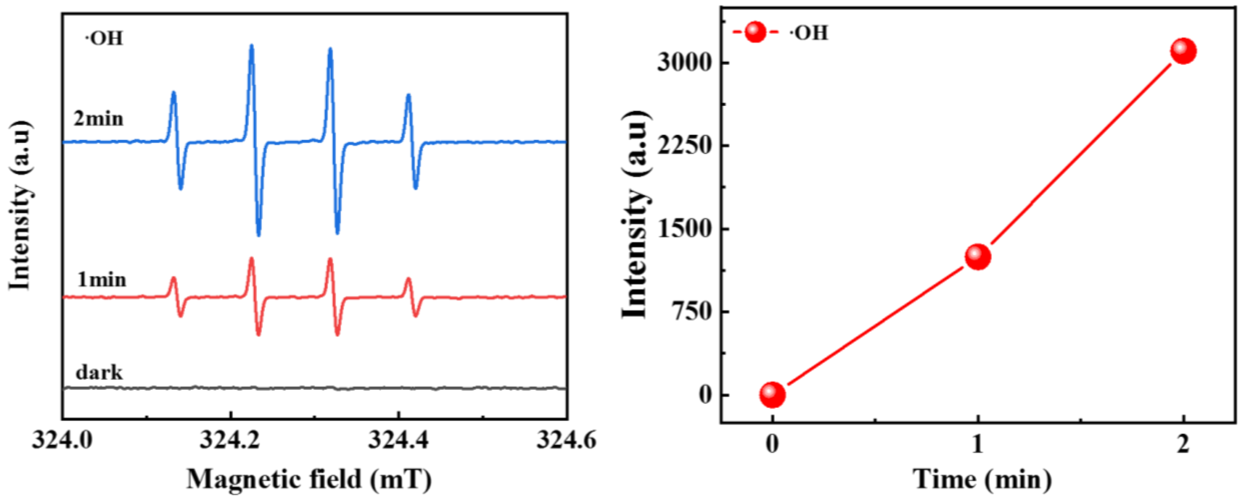

Figure S34. ESR spectra of free radicals trapped by DMPO (left) in the reaction with ***m*CB-MOF-2** in the dark and under UV light irradiation in water for 1 and 2 min and $OH^{\bullet}$ generation rate (right).

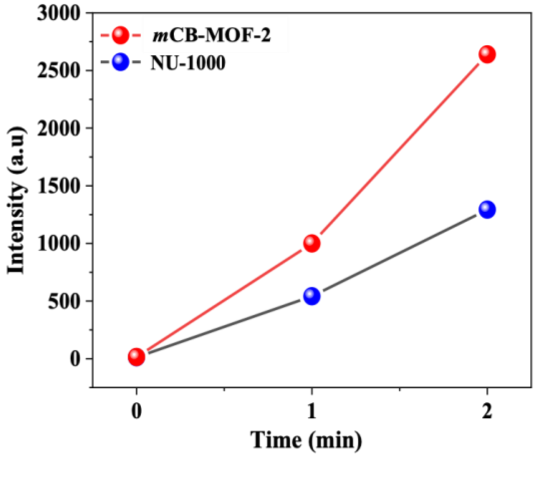

Figure S35. Comparison of $^{1}O_2$ generation rates on NU-1000 and ***m*CB-MOF-2**.

References

1. Juanhuix, J.; Gil-Ortiz, F.; Cuni, G.; Colldelram, C.; Nicolas, J.; Lidon, J.; Boter, E.; Ruget, C.; Ferrer, S.; Benach, J., Developments in optics and performance at BL13-XALOC, the macromolecular crystallography beamline at the Alba Synchrotron. *Journal of Synchrotron Radiation* **2014,** *21* (4), 679-689.
2. Dolomanov, O. V.; Bourhis, L. J.; Gildea, R. J.; Howard, J. A. K.; Puschmann, H., OLEX2: a complete structure solution, refinement and analysis program. *Journal of Applied Crystallography* **2009,** *42* (2), 339-341.
3. Sheldrick, G., Crystal structure refinement with SHELXL. *Acta Crystallographica Section C* **2015,** *71* (1), 3-8.
4. Kieffer, J.; Karkoulis, D., PyFAI, a versatile library for azimuthal regrouping. *Journal of Physics: Conference Series* **2013,** *425* (20), 202012.
5. Fox, M. A., Icosahedral carborane derivatives. *Durham theses, Durham University.* **1991**.
6. Pankajakshan, A.; Sinha, M.; Ojha, A. A.; Mandal, S., Water-stable nanoscale zirconium-based metal–organic frameworks for the effective removal of glyphosate from aqueous media. *ACS omega* **2018,** *3* (7), 7832-7839.
7. VandeVondele, J.; Krack, M.; Mohamed, F.; Parrinello, M.; Chassaing, T.; Hutter, J., Quickstep: Fast and accurate density functional calculations using a mixed Gaussian and plane waves approach. *Comput. Phys. Commun.* **2005,** *167* (2), 103-128.
8. Goedecker, S.; Teter, M.; Hutter, J., Separable dual-space Gaussian pseudopotentials. *Physical Review B* **1996,** *54* (3), 1703.
9. Hartwigsen, C.; Gœdecker, S.; Hutter, J., Relativistic separable dual-space Gaussian pseudopotentials from H to Rn. *Physical Review B* **1998,** *58* (7), 3641.
10. Krack, M.; Parrinello, M., All-electron ab-initio molecular dynamics. *Phys. Chem. Chem. Phys.* **2000,** *2* (10), 2105-2112.
11. VandeVondele, J.; Hutter, J., Gaussian basis sets for accurate calculations on molecular systems in gas and condensed phases. *The Journal of chemical physics* **2007,** *127* (11), 114105.
12. Perdew, J. P.; Burke, K.; Ernzerhof, M., Generalized gradient approximation made simple. *Phys. Rev. Lett.* **1996,** *77* (18), 3865.
13. Grimme, S.; Antony, J.; Ehrlich, S.; Krieg, H., A consistent and accurate ab initio parametrization of density functional dispersion correction (DFT-D) for the 94 elements H-Pu. *The Journal of chemical physics* **2010,** *132* (15), 154104.
14. Börjesson, E.; Torstensson, L., New methods for determination of glyphosate and (aminomethyl)phosphonic acid in water and soil. *Journal of Chromatography A* **2000,** *886* (1), 207-216.
15. Yamaguchi, N. U.; Bergamasco, R.; Hamoudi, S., Magnetic MnFe2O4–graphene hybrid composite for efficient removal of glyphosate from water. *Chemical Engineering Journal* **2016,** *295*, 391-402.
16. Zheng, T.; Sun, Y.; Lin, Y.; Wang, N.; Wang, P., Study on preparation of microwave absorbing MnOx/Al2O3 adsorbent and degradation of adsorbed glyphosate in MW–UV system. *Chemical Engineering Journal* **2016,** *298*, 68-74.
17. Mayakaduwa, S.; Kumarathilaka, P.; Herath, I.; Ahmad, M.; Al-Wabel, M.; Ok, Y. S.; Usman, A.; Abduljabbar, A.; Vithanage, M., Equilibrium and kinetic mechanisms of woody biochar on aqueous glyphosate removal. *Chemosphere* **2016,** *144*, 2516-2521.

18. Carneiro, R. T.; Taketa, T. B.; Neto, R. J. G.; Oliveira, J. L.; Campos, E. V.; de Moraes, M. A.; da Silva, C. M.; Beppu, M. M.; Fraceto, L. F., Removal of glyphosate herbicide from water using biopolymer membranes. *Journal of environmental management* **2015,** *151*, 353-360.
19. Milojević-Rakić, M.; Janošević, A.; Krstić, J.; Vasiljević, B. N.; Dondur, V.; Ćirić-Marjanović, G., Polyaniline and its composites with zeolite ZSM-5 for efficient removal of glyphosate from aqueous solution. *Microporous mesoporous materials* **2013,** *180*, 141-155.
20. Khoury, G. A.; Gehris, T. C.; Tribe, L.; Sánchez, R. M. T.; dos Santos Afonso, M., Glyphosate adsorption on montmorillonite: An experimental and theoretical study of surface complexes. *Applied Clay Science* **2010,** *50* (2), 167-175.
21. Hu, Y.; Zhao, Y.; Sorohan, B., Removal of glyphosate from aqueous environment by adsorption using water industrial residual. *Desalination* **2011,** *271* (1-3), 150-156.
22. Khenifi, A.; Derriche, Z.; Mousty, C.; Prévot, V.; Forano, C., Adsorption of glyphosate and glufosinate by Ni2AlNO3 layered double hydroxide. *Applied Clay Science* **2010,** *47* (3-4), 362-371.
23. Jonsson, C. M.; Persson, P.; Sjöberg, S.; Loring, J. S., Adsorption of glyphosate on goethite (α-FeOOH): surface complexation modeling combining spectroscopic and adsorption data. *Environmental science technology* **2008,** *42* (7), 2464-2469.
24. Li, F.; Wang, Y.; Yang, Q.; Evans, D. G.; Forano, C.; Duan, X., Study on adsorption of glyphosate (N-phosphonomethyl glycine) pesticide on MgAl-layered double hydroxides in aqueous solution. *Journal of hazardous materials* **2005,** *125* (1-3), 89-95.
25. Zhu, X.; Li, B.; Yang, J.; Li, Y.; Zhao, W.; Shi, J.; Gu, J., Effective adsorption and enhanced removal of organophosphorus pesticides from aqueous solution by Zr-based MOFs of UiO-67. *ACS applied materials & interfaces* **2015,** *7* (1), 223-231.
26. Yang, Q.; Wang, J.; Chen, X.; Yang, W.; Pei, H.; Hu, N.; Li, Z.; Suo, Y.; Li, T.; Wang, J., The simultaneous detection and removal of organophosphorus pesticides by a novel Zr-MOF based smart adsorbent. *Journal of Materials Chemistry A* **2018,** *6* (5), 2184-2192.
27. Yang, Q.; Wang, J.; Zhang, W.; Liu, F.; Yue, X.; Liu, Y.; Yang, M.; Li, Z.; Wang, J., Interface engineering of metal organic framework on graphene oxide with enhanced adsorption capacity for organophosphorus pesticide. *Chemical Engineering Journal* **2017,** *313*, 19-26.
28. Artikis, E.; Brooks, C. L., Modeling pH-Dependent NMR Chemical Shift Perturbations in Peptides. *Biophysical Journal* **2019,** *117* (2), 258-268.
29. CHEBI:57433 - sarcosine zwitterion. https://www.ebi.ac.uk/chebi/chebiOntology.do?chebiId=CHEBI%3A57433 (accessed November 11).
30. Ciftja, A. F.; Hartono, A.; Svendsen, H. F., Selection of Amine Amino Acids Salt Systems for CO2 Capture. *Energy Procedia* **2013,** *37*, 1597-1604.
